# Continuous Culture Adaptation of *Methylobacterium extorquens* AM1 and TK 0001 to Very High Methanol Concentrations

**DOI:** 10.3389/fmicb.2019.01313

**Published:** 2019-06-20

**Authors:** Sophia Belkhelfa, David Roche, Ivan Dubois, Anne Berger, Valérie A. Delmas, Laurence Cattolico, Alain Perret, Karine Labadie, Aude C. Perdereau, Ekaterina Darii, Emilie Pateau, Véronique de Berardinis, Marcel Salanoubat, Madeleine Bouzon, Volker Döring

**Affiliations:** Génomique Métabolique, Genoscope, Institut François Jacob, CEA, CNRS, Université d’Évry, Université Paris-Saclay, Évry, France

**Keywords:** methylotrophic bacteria, directed evolution, continuous culture, genomics, transcriptomics, lactate

## Abstract

The bio-economy relies on microbial strains optimized for efficient large scale production of chemicals and fuels from inexpensive and renewable feedstocks under industrial conditions. The reduced one carbon compound methanol, whose production does not involve carbohydrates needed for the feed and food sector, can be used as sole carbon and energy source by methylotrophic bacteria like *Methylobacterium extorquens* AM1. This strain has already been engineered to produce various commodity and high value chemicals from methanol. The toxic effect of methanol limits its concentration as feedstock to 1% v/v. We obtained *M. extorquens* chassis strains tolerant to high methanol via adaptive directed evolution using the GM3 technology of automated continuous culture. Turbidostat and conditional medium swap regimes were employed for the parallel evolution of the recently characterized strain TK 0001 and the reference strain AM1 and enabled the isolation of derivatives of both strains capable of stable growth with 10% methanol. The isolates produced more biomass at 1% methanol than the ancestor strains. Genome sequencing identified the gene *metY* coding for an *O*-acetyl-L-homoserine sulfhydrylase as common target of mutation. We showed that the wildtype enzyme uses methanol as substrate at elevated concentrations. This side reaction produces methoxine, a toxic homolog of methionine incorporated in polypeptides during translation. All mutated *metY* alleles isolated from the evolved populations coded for inactive enzymes, designating *O*-acetyl-L-homoserine sulfhydrylase as a major vector of methanol toxicity. A whole cell transcriptomic analysis revealed that genes coding for chaperones and proteases were upregulated in the evolved cells as compared with the wildtype, suggesting that the cells had to cope with aberrant proteins formed during the adaptation to increasing methanol exposure. In addition, the expression of ribosomal proteins and enzymes related to energy production from methanol like formate dehydrogenases and ATP synthases was boosted in the evolved cells upon a short-term methanol stress. D-lactate production from methanol by adapted cells overexpressing the native D-lactate dehydrogenase was quantified. A significant higher lactate yield was obtained compared with control cells, indicating an enhanced capacity of the cells resistant to high methanol to assimilate this one carbon feedstock more efficiently.

## Introduction

Methanol is a highly available industrial compound that can be produced from the greenhouse gas carbon dioxide by chemical or electrolytic reduction processes (Ganesh, 2014). A growing number of methanol based biotechnological production pathways are developed as alternatives to industrial fermentations relying on sugar as feedstock (Schrader et al., 2009; Bennett et al., 2018). Among methylotrophic organisms capable of growing on methanol as the sole carbon and energy source, the facultative methylotrophic alpha-proteobacterium *Methylobacterium extorquens* AM1 is widely used as catalyst and has been engineered for the production of a variety of value-added chemicals or biofuels (Hu and Lidstrom, 2014; Hu et al., 2016; Zhu et al., 2016).

The central carbon and energy metabolism of *M. extorquens* AM1, which belongs to the serine-cycle methylotrophs, has been studied in detail (Chistoserdova et al., 2003; Ochsner et al., 2015). Genomic sequences (Vuilleumier et al., 2009; Marx et al., 2012) and metabolic models (Marx et al., 2005; Peyraud et al., 2011) are also available for this and other members of the *Methylobacterium* genus. With the objective to construct platform strains for the optimal use in biotechnological applications and to answer fundamental questions of evolution, attempts have been made to optimize methanol assimilation through rewiring central steps of formaldehyde conversion (Carroll et al., 2015) or to broaden the set of one carbon (C1) compounds used as substrates (Michener et al., 2014).

A limitation of industrial scale methanol fermentation is the low tolerance of most of the methylotrophic organisms toward methanol. The toxicity of methanol is, at least partially, attributable to its properties as organic solvent (Busby et al., 1999; Hwang et al., 2011). *M. extorquens* AM1 has a growth maximum with 1% (v/v) methanol. Low solvent tolerance is a common hurdle of biotechnology, notably for processes of biofuel production. Since solvent stressors usually have pleiotropic effects on cell integrity and proliferation (Stephanopoulos et al., 2004), directed evolution rather than rational design approaches are followed to select for enhanced tolerance.

Numerous reports relay the adaptation of the natural producer strain *Saccharomyces cerevisiae* to higher ethanol tolerance (Ma and Liu, 2010). Likewise, mutants of the reference strain *Escherichia coli* were obtained exhibiting resistance to ethanol (Yomano et al., 1998), n-butanol (Rutherford et al., 2010; Reyes et al., 2011, 2013), and isobutanol (Atsumi et al., 2010).

Phenotypic analysis of resistant cells, including genomic, transcriptomic, and metabolomics studies, often revealed complex patterns of adaptations, which depended on the nature of the solvent. Changes included the lipid content of cell membranes, the enhanced synthesis of peptidoglycan precursors, and overexpression of chaperones or oxidative stress responses. In some cases, stress resistance could be attributed to point mutations in particular genes. This was the case for *M. extorquens* AM1 mutants growing with increased n-butanol concentrations, where a potassium-proton antiporter was found to partially account for the adaptive phenotype (Hu et al., 2016).

In addition to direct effects of methanol as alcoholic solvent, the molecule can exhibit indirect toxicity due to its metabolic conversion to formaldehyde and formate (Liesivuori and Savolainen, 1991; Oyama et al., 2002; Chen et al., 2016). Resistance of *Bacillus methanolicus* toward high methanol was found to depend on the overexpression of the genes of the ribulose monophosphate cycle converting formaldehyde into biomass (Jakobsen et al., 2006). Besides toxicity via oxidation, for some strains experimental evidence pointed to the conversion of methanol at high concentrations to the toxic methionine analog methoxine (Leßmeier and Wendisch, 2015; Schotte et al., 2016).

In this work, parallel experiments of directed evolution were performed in GM3 continuous culture automatons (Marlière et al., 2011) to adapt the recently characterized *M. extorquens* strain TK 0001 (Belkhelfa et al., 2018) and strain AM1 to growth on methanol concentrations of up to 10% (v/v). Genome sequencing of isolates of both lineages were conducted to identify common mutations potentially relevant for the methanol tolerant phenotype. The *metY* gene coding for *O*-acetyl-L-homoserine sulfhydrylase was found to harbor missense mutations in all isolates sequenced. A causal genotype-phenotype relation was experimentally demonstrated for this mutated locus. Furthermore, a transcriptomic analysis revealed differences in expression patterns of wildtype and methanol tolerant cells provoked by methanol stress. Finally, quantifying D-lactate production by an engineered methanol resistant TK 0001 mutant further demonstrated the usefulness of directed evolution for the selection of industrial production chassis.

## Materials and Methods

### Bacterial Strains, Culture Media, and Growth Assays

The strains *M. extorquens* AM1 (DSM 1338) and TK 0001 (DSM 1337) were grown at 30°C on a standard mineral (SM) medium (DSMZ n°1629) supplemented with methanol at different concentrations (v/v) as the sole carbon source. For growth curve experiments, a Microbiology Reader Bioscreen C apparatus (Thermo Fisher Scientific) was used consisting of a thermostatic incubator and a culture growth-monitoring device (OD reader). Precultures in SM medium supplemented with 1% methanol (v/v) were diluted in growth medium to reach an OD_600__nm_ of 0.01; 200 μL aliquots of the cell suspensions were distributed into honeycomb 100-wells plates. The plates were incubated at 30°C under continuous agitation. Bacterial growth was followed by recording optical densities at 600 nm (OD_600__nm_) every 15 min for 72 h if not otherwise stated. Each experiment was performed in triplicate and repeated twice. In the case of growth experiments of prolonged duration (over 5 days), the cultures were performed in 125 mL flasks with a starting volume of 25 mL and incubated at 30°C with constant agitation. OD_600__nm_ of aliquots sampled every 3 h during working hours was measured in an optical spectrophotometer (Beckmann).

### Continuous Cultures

Evolution experiments in continuous culture were carried out using automaton-driven GM3 fluidic self-cleaning cultivation devices. This device automatically dilutes growing cell suspensions with nutrient medium by keeping the culture volume constant. A continuous flow of sterile air through the culture vessel ensures constant aeration and counteracts cell sedimentation. Different growth regimes can be programmed.

#### Turbidostat

This cultivation regime enables the selection of optimized growth in permissive conditions. Every 10 min, the optical density of the culture is automatically measured and compared to a fixed threshold (OD_880__nm_ value of 0.4). When the measured OD_880__nm_ exceeds the threshold, a pulse of fresh nutrient medium is injected into the culture and the same volume of used culture discarded. The dilutions ensure that the biomass in the vessel remains constant and that the bacteria grow at their maximal growth rate.

#### Medium Swap

This regime enables gradual adaptation of a bacterial population to grow in a non-permissive or stressing medium. The growing culture can be diluted by either permissive or stressing medium. The choice between the two dilution media depends on the turbidity of the culture with respect to a set OD_880__nm_ threshold (OD_880__nm_ value of 0.4). When the OD_880__nm_ exceeds the threshold, a pulse of stressing medium is injected; otherwise a pulse of permissive medium is injected. Dilutions are triggered every 10 min with a fixed volume of medium, thus imposing a generation time on the cell population.

A population of growing cells of both strains *M. extorquens* TK 0001 and AM1 was injected in an automated GM3 cultivation device at 30°C. The composition of the permissive and stressing media used during the evolution experiments and set generation time under medium swap regime were as follows:

• Selection of growth at 5% methanol: permissive medium: SM medium + 1% methanol – stressing medium: SM medium + 5% methanol – generation time 10 h for TK 0001 and 8 h for AM1.
• Selection of growth at 7% methanol: permissive medium: SM medium + 5% methanol – stressing medium: SM medium + 7% methanol – generation time 13 h for TK 0001 and 7 h 30 for AM1.
• Growth adaptation cultures were performed in the turbidostat mode using SM medium supplemented with 8, 9, and 10% methanol successively.

### Whole Genome Sequencing and Mutational Analysis

The genomes of six isolates obtained from GM3 cultures sampled at each adaptation step to increasing methanol concentrations were sequenced at CEA/Genoscope. Genomic DNA was extracted using the GenElute bacterial genomic DNA kit (Sigma, NA2110). One hundred nanograms of DNA was fragmented into 450-bp mean size fragments by Covaris E220 (Covaris, Inc., Woburn, MA, United States). The fragments were end-repaired, A-tailed at the 3′-end and ligated to Illumina compatible adapters using the NEBNext DNA Sample Prep Master Mix kit (New England BioLabs) with a “on beads” protocol. Ligation products were purified and amplified using the Kapa Hifi HotStart NGS library amplification kit (Kapa Biosystems KK2611), followed by 0.6× AMPure XP purification. Libraries traces were validated on Agilent 2100 Bioanalyzer (Agilent Technologies, United States) and quantified by qPCR using the KAPA Library Quantification Kit (KapaBiosystems) on a MxPro instrument (Agilent Technologies, United States). Libraries were sequenced using 100-bp paired-end reads on an Illumina MiSeq.

High-throughput sequencing (HTS) data were analyzed using the PALOMA bioinformatic pipeline implemented in the MicroScope platform (Médigue et al., 2017). In a first step, reads were mapped onto the *M. extorquens* TK 0001 or *M. extorquens* AM1 genome reference using the SSAHA2 package (v.2.5.1) (Ning, 2001). Only unique matches having an alignment score equal to at least half of their length were retained as seeds for full Smith-Waterman realignment (Smith and Waterman, 1981) with a region extended on both sides by five nucleotides of the reference genome. All computed alignments then were screened for discrepancies between read and reference sequences and a score based on coverage, allele frequency, quality of bases, and strand bias was computed for each detected event to assess its relevance. Finally the mutation (SNPs and small indels) with a score superior to 0.8 and with at least five supporting reads were kept.

### Transcriptome Analysis

Cultures of exponentially growing cells of the wildtype strain *M. extorquens* TK 0001 and the evolved strain G4105 in SM supplemented with 1% methanol were split in two parts. One part was cultivated further in the same condition and the second part was cultivated after addition of methanol to reach 5% methanol final concentration. 50 mL of cell suspension were retrieved from each culture after 5 min and 3 h of cultivation. The whole experiment was performed in triplicate. Total RNA of all samples was extracted using MBER (Ambion 46_6036) and Trizol reagent (Sigma, 93289), and was treated twice with Turbo DNAse (Thermo Fisher Scientific) for 30 min at 37°C. Small RNAs shorter than 200 bp were removed using Zymoclean (Zymo Research R1015). Total RNA (5 μg) were firstly depleted of bacterial rRNA using Ribo-Zero rRNA Removal Kit (bacteria) (Illumina MRZMB126), then purified using the RNA Clean and Concentrator kit (Zymo Research). RNA-Seq library preparations were then carried out from 100 ng of bacterial ribo-depleted RNA using the TruSeq Stranded mRNA kit (Illumina, San Diego, CA, United States) without poly-A selection. After library profile analysis by Agilent 2100 Bioanalyzer (Agilent Technologies, United States) and qPCR quantification (MxPro, Agilent Technologies, United States), libraries were sequenced on an Illumina HiSeq 4000 sequencer with paired-end 150-bp reads.

Transcriptome sequencing data were analyzed using the TAMARA bioinformatics pipeline implemented in the MicroScope platform (Médigue et al., 2017). In a first step, reads were mapped onto the *M. extorquens* TK 0001 genome reference (BWA-MEM v.0.7.4) (Li, 2013). An alignment score equal to at least half of the read was required for a hit to be retained. To lower the false positive discovery rate, the SAMtools (v.0.1.8) (Li et al., 2009) were then used to extract reliable alignments from SAM formatted files. The number of reads matching each genomic object (GO) present on the reference genome was subsequently computed with the toolset BEDTools (v.2.10.1) (Quinlan and Hall, 2010). Finally, the Bioconductor-DESeq package [v.1.4.1 (Anders and Huber, 2010)] was applied with default parameters to analyze raw counts data and test for differential expression between conditions. Gene expression fold changes between two experimental conditions were considered significant if the log_2_(fold change) was greater than 2 or less than −2 and the calculated *p*-adjusted value was smaller than 0.05.

### MetY Overexpression

The wildtype and mutated forms of the *metY* gene were PCR-amplified using the following primers: forward primer 5739 5′-AAAGAAGGAGATAGGATCATGCATCATCACCATCACC ATTCGGACCAGACGCCCGCTTCAGTCTACG and reverse primer 5743 5′-GTGTAATGGATAGTGATCTTAGGCCGCCCG CGCCAGCGTT. The resulting fragments were inserted into a pET22b(+) vector (Novagen) modified for ligation independent cloning (Bastard et al., 2013). The sequence of all inserts was checked by Sanger sequencing. The resulting plasmids were introduced into *E. coli* BL21 (DE3) by chemical transformation. Standard methods were used for cell culture, induction of gene expression, cell extract preparation and purification of 6xHis-tagged recombinant proteins. The same overexpression protocol was used for the wildtype and mutated *metY* genes. Purified enzymes were stored at -80°C. The samples were analyzed by SDS-PAGE using the NuPAGE system. Protein concentrations were determined using Bradford method with bovine serum albumin as the standard (Bio-Rad).

### MetY Biochemical Assay

MetY kinetic parameters were determined by a coupled enzymatic assay using an acetic acid assay kit that uses pyruvate kinase and lactate dehydrogenase for the detection of acetic acid (Megazyme). The oxidation of NADH concomitant with *O*-acetyl-L-homoserine (Toronto Research Chemicals) transhydroxylation with either sodium sulfide (Sigma-Aldrich, 431648) or methanol (Sigma-Aldrich, 34860) was monitored at 340 nm in a SAFAS UVmc2 double-beam spectrophotometer. All kinetic parameters were determined from duplicate experiments by non-linear analysis of initial rates with SigmaPlot version 9.0 (Systat Software). Enzymatic reactions were performed at RT in 100 μL total volume reaction at pH 7.4 containing NADH at 4 mM and initiated by the addition of 2 ng of the purified enzyme. For the wildtype protein assay, we varied the concentration of one substrate and set the other substrate at a saturating concentration (*O*-acetyl-L-homoserine versus sulfide or methanol). The mutated proteins were tested with the saturating concentrations determined for the wildtype protein as described above.

### Fatty Acid Content Characterization

Whole-cell fatty acid content was characterized for both wildtype strains *M. extorquens* TK 0001 and AM1, as well as fatty acid content of the evolved isolates G4105, G4521, G4609, and G4706. One hundred milligrams of exponentially growing cells in SM medium supplemented with 1% methanol were pelleted by centrifugation. Fatty acid analyses were carried out by the Identification Service of the DSMZ (Braunschweig, Germany^1^).

### Lactate Production Measurement

#### Metabolome Preparation

Cells were grown in 2.5 ml at 30°C. Metabolite extraction was adapted from the Metabolomics Service Protocols from the University of Glasgow^2^. A saturated overnight culture was diluted in fresh medium to reach OD_600__nm_ = 0.05. 2.5 ml of cell suspension were distributed in each of 23 wells of a 24 deep-well plate (Whatman; reference 7701-5110). The cells were further grown to OD_600__nm_ between 2.0 and 3.2 (log phase) in a shaking incubator (Climo Shaker ISF1-X Kühner). The plate was then centrifuged at 2700 *g* at 4°C for 10 min and the supernatant removed. The cell pellets were suspended in 200 μl of water/methanol/acetonitrile (1:3:1 ratio) and placed in a cold bath (-80°C) composed of dry ice and ethanol. After cell freezing, the mixtures were let at 25°C to complete unfreezing of the cells. This procedure was repeated twice to obtain complete breakage of the cells. The lysates were rocked on a shaker for 1 h at 4°C then centrifuged at 5000 *g* at 4°C for 10 min. The supernatants were dried and stored at -80°C. Before LC/MS analysis, the metabolites were resuspended in 20 μl water and 42 μl of acetonitrile: 10 mM ammonium acetate (80:20), centrifuged at 5,000 *g* at 4°C for 10 min. The supernatants were filtered on 0.22 μm (PTFE, Acroprep Advance, Pall).

Lactate detection was performed on a Thermo Fisher Scientific^TM^ Dionex^TM^ UltiMate^TM^ 3000 Rapid Separation (3000RS) liquid chromatography (LC) system (Thermo Fisher Scientific, Courtaboeuf, France) coupled to ultra-high resolution Orbitrap Elite hybrid mass spectrometer (Thermo Fisher Scientific, Courtaboeuf, France).

#### High-Pressure Liquid Chromatographic Conditions

Chromatographic separation was carried out on a Sequant ZIC^®^pHILIC column (5 μm, 2.1 mm × 100 mm, Merck, Darmstadt, Germany) thermostated at 40°C with a mobile phase flow rate of 200 μl.min^–1^. Aqueous solution of 10 mM ammonium acetate was used as phase A and acetonitrile as phase B. The following elution conditions were applied: 1 min isocratic step at 80% of solvent B, 7 min linear gradient from 80 to 40% of solvent B, 2.5 min isocratic elution at 40% of solvent B, return to the initial composition (80% of solvent B) in 2 min, and re-equilibrated under these conditions for 10 min. Five microliters were injected.

#### Mass Spectrometry Analysis

Mass spectrometer fitted with a heated electrospray ionization source (HESI) was operated in the negative ionization mode. Electrospray voltage was set at −4 kV. Sheath gas and auxiliary gas flow rates were set at 60 arbitrary units (a.u.) and 44 a.u., respectively, and the drying gas temperature was set at 275°C. The mass resolution power of the detector was 60,000 m/Δm (full width at half maximum, FWHM at m/z 400). Mass spectra were acquired over an m/z range from m/z 50 up to m/z 1000. Raw data were analyzed using the Qual-browser module of Xcalibur version 2.2 (Thermo Fisher Scientific, Courtaboeuf, France).

#### Quantification

A calibration curve for lactate was required to calculate its concentration. We prepared calibration solutions containing 0, 1, 2, 4, 5, 8, 10, 20, 25, 50, and 100 μM sodium lactate. These solutions were prepared in an initial volume of 200 μl of water/methanol/acetonitrile (1:3:1 ratio) and were treated as the cell pellets (see above) before LC/MS injection. Samples were analyzed in the negative mode. The peak areas from Extracted Ion Chromatograms (EIC) of lactate were integrated. Metabolomes from 23 independent 2.5 ml cultures for each experimental condition were used to estimate the metabolite concentration. The lyophilized metabolomes were suspended with 62 μl 80% acetonitrile and 20% 10 mM ammonium acetate. Samples were filtered on 0.22 μm prior to injection.

#### Statistical Analysis

Statistical analyses were performed using XLSTAT (Addinsoft). The normal distribution of lactate values was estimated using the Shapiro–Wilk test. The non-parametric Kruskal–Wallis test was used to determine if lactate concentration was statistically different between the four strains (*p* < 0.05). Multiple pairwise comparisons were conducted using the Dunn method with Bonferroni correction.

### Methoxine Detection

For the detection of methoxine, an enzymatic reaction was performed in 1800 μL volume of Tris/HCl buffer at pH 8.0 containing 5 mM of *O*-acetyl-L-homoserine and 3 M of methanol. The reaction was initiated by the addition of 64 μg of purified wildtype MetY protein and 200 μl samples were withdrawn at the initiation of the reaction and after 20, 40, 60, and 120 min. The samples were filtered (VWR PES 3K, 14000 g) to stop the reaction and remove the enzyme.

Chromatographic separation was carried out on Sequant ZIC^®^pHILIC column (5 μm, 150 mm × 2.1 mm, Merck, Darmstadt, Germany) thermostated at 40°C with a mobile phase flow rate of 200 μl.min^–1^. Aqueous solution of 10 mM ammonium acetate was used as phase A and acetonitrile as phase B. The gradient started at 80% B for 2 min, followed by a linear gradient from 80 to 40% B at 20 min and remained 3 min at 40% B. The system returned to the initial composition in 2 min, and re-equilibrated under these conditions for 12 min. Five microliters were injected. For the mass spectrometry analysis, the same protocol as for lactate determination was used.

## Results

### Selection of *M. extorquens* TK 0001 Strains Resistant to High Methanol

**FIGURE 1:**
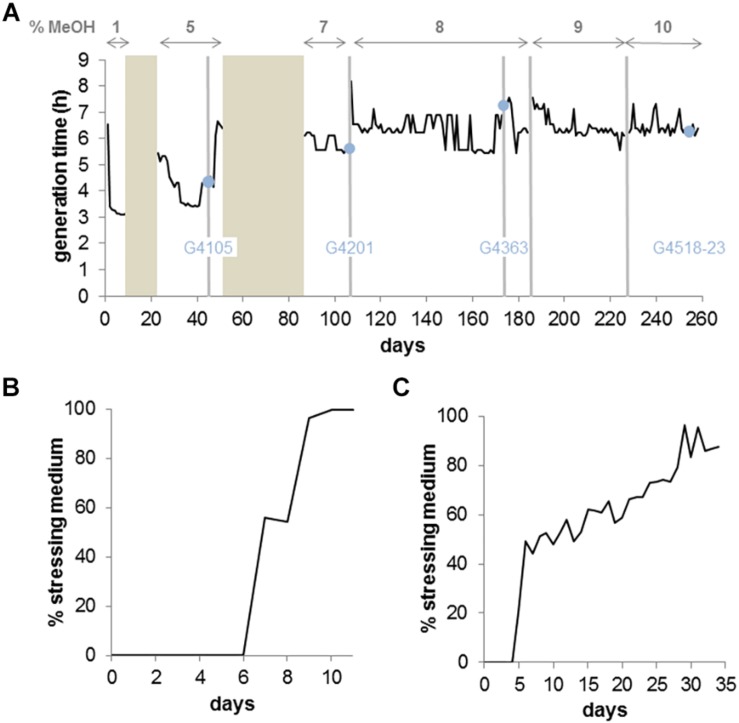
Continuous culture adaptation of *M. extorquens* TK 0001 bacteria to increasing methanol concentrations in a GM3 device. **(A)** Growth rate recorded for 260 days of a bacterial population cultivated on augmenting methanol concentrations. Bronze areas correspond to periods of cultivation under medium-swap regime. Black lines show the average daily generation time during cultivation periods under turbidostat regime. The methanol concentration of the culture medium applied for each evolution step (delimited by vertical gray bars) is indicated on top of the graph. Blue dots indicate time points of sampling and isolation of intermediate strains. The designation of the strain inoculated for the following adaptation step is indicated. **(B)** Evolutionary kinetics of adaptive growth on minimal medium 5% methanol of *M. extorquens* TK 0001 wildtype bacteria cultivated under medium-swap regime. A generation time of 10 h was set by the volume of the medium pulses injected at regular time intervals. The daily ratio of stressing medium pulses is plotted as a function of time. **(C)** Evolutionary kinetics of adaptive growth on minimal medium 7% methanol of the 5% methanol-adapted strain G4105 cultivated under medium-swap regime. A generation time of 13 h was set. The daily ratio of stressing medium pulses is plotted as a function of time.

We adapted *M. extorquens* TK 0001, a recently characterized strain showing strong sequence homologies to *M. extorquens* AM1 but lacking plasmids (Belkhelfa et al., 2018), to growth on increasing methanol concentrations (Figure 1). As previously reported for several *M. extorquens* strains, the optimum growth of strain TK 0001 was obtained with 1% methanol (v/v) (Supplementary Figure S1). We used the self-cleaning GM3 cultivation device enabling automated long term strain adaptations under controlled growth conditions (Marlière et al., 2011). The adaptation of *M. extorquens* TK 0001 was initiated with a culture at 1% methanol growing under a turbidostat regime where fastest growing cells are selected by diluting the culture with fresh medium each time a cell density threshold is passed (Figure 1A). To select for methanol resistance, the culture was grown in a conditional medium swap regime (Marlière et al., 2011) (see section “Continuous Cultures”). The relaxing medium contained, as did the initial turbidostat culture, 1% methanol, while the stressing medium contained 5% methanol (Figure 1B). After an adaptation period of 11 days under this regime, the ratio of stressing to relaxing dilution pulses reached 100%, signifying that the cell population grew with 5% methanol. After a short period (26 days) of growth with the 5% methanol medium in turbidostat to stabilize the cells and lower the generation time of the culture, isolated colonies were obtained from the adapted cell population on semi-solid medium containing 5% methanol. Six isolates were finally chosen from the best growing colonies for further analysis. One of the isolates, strain G4105, was used to inoculate a GM3 culture for further adaptation to 7% methanol. Again, a medium swap regime was employed, oscillating between 5 and 7% methanol for the relaxing and stressing medium, respectively (Figure 1C). Growth of the culture with 7% methanol was obtained after 25 days and followed by a turbidostat growth period of 19 days to consolidate the culture. At this time point, colonies were isolated on solid medium with 7% methanol and six of them were chosen from the best growers. Isolate G4201 was then adapted to growth at 8% methanol. Given the extremely high solvent concentration (1.9 M) in the cultures, adaptation was performed with an increment of 1% in turbidostat. After 65 days, six isolates were obtained showing growth with 8% methanol on solid medium. A culture from one of the isolates, strain G4363, was further adapted to growth with 9% and finally 10% methanol under turbidostat regimes (Figure 1A). Again, six isolates were chosen from this final population of the evolution experiment for further analysis (for nomenclature of strains and evolved isolates, refer to Supplementary Table S1).

**FIGURE 2:**
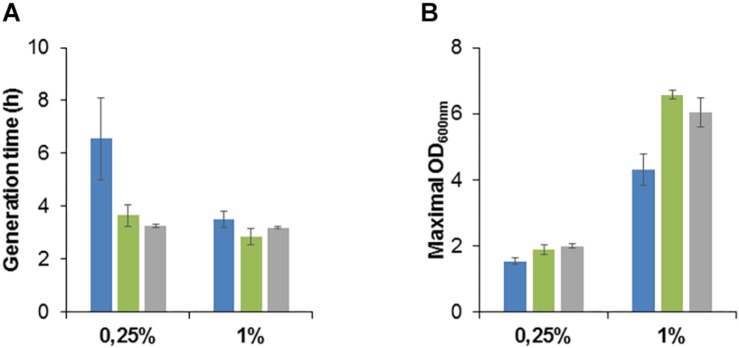
Growth phenotype of *M. extorquens* TK 0001 wildtype strain (blue bars) and methanol evolved isolates G4105 (green bars) and G4521 (gray bars). Generation time during exponential growth phase **(A)** and maximal OD_600__nm_ as an estimate of biomass yield **(B)** were determined after cultivation on minimal medium supplemented with 0.25 or 1% methanol (v/v) in shaking flasks. Each result is the mean of triplicates; ± standard deviation is indicated.

**FIGURE 3:**
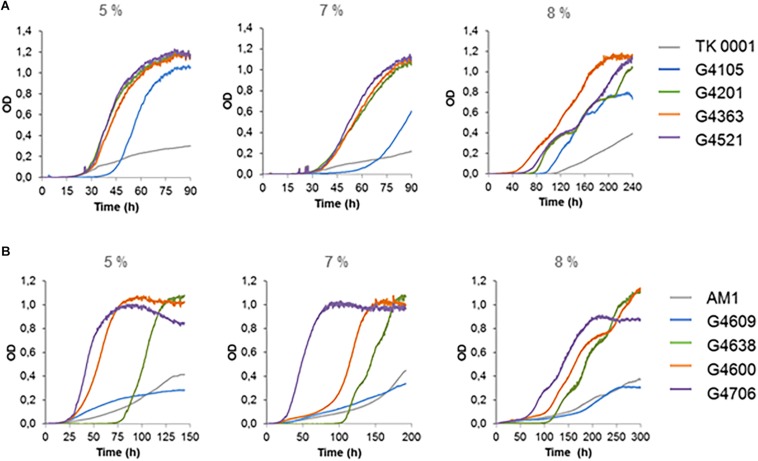
Plate reader growth profiles of *M. extorquens* TK 0001 wildtype strain and evolved isolates **(A)** and of *M. extorquens* AM1 wildtype strain and evolved isolates **(B)** on SM media supplemented with 5, 7, and 8% methanol (v/v). Growth experiments were performed in triplicates; measure deviations between samples were less than 5%.

We studied the growth phenotype of the isolated methanol-resistant TK 0001 strains. The generation time during exponential growth was determined for the strains G4105 (5% methanol adaptation) and G4521 (10% methanol adaptation) at permissive methanol concentrations. Figure 2A shows a significant reduction of the doubling time at 0.25% methanol for both adapted strains as compared to the wildtype. The growth acceleration was much less pronounced at 1% methanol. Conversely, the determination of the maximal OD_600 nm_ as an indicator of the maximal biomass yield revealed a considerable gain for the adapted strains at 1% methanol, but only a small effect at 0.25% (Figure 2B). No significant differences neither in growth rate nor yield was found between the two adapted strains tested. We further examined whether the growth phenotypes of the resistant cells differ at high methanol concentrations (Figure 3A). At 5% methanol, the adapted strains G4201 (adaptation to 7%), G4363 (adaptation to 8%), and G4521 (adaptation to 10%) showed overlapping growth curves. Growth of strain G4105 (adaptation to 5%) was very similar but lagged for about 15 h under these conditions. At 7 and 8% methanol, differences in growth behavior between the three strains adapted to higher methanol became apparent, with strain G4521 exhibiting fastest growth. These results suggest that the cell population continued to evolve until growth at 10% methanol under turbidostat regime.

### Comparative Genomic Analysis of Methanol Resistant Isolates

To identify specific mutations which occurred during the adaptation of the cell populations to growth on high methanol, genomic sequencing was performed for the six isolates obtained from the cultures adapted to 5% (culture MEM5), 7% (culture MEM7), 8% (culture MEM8), and 10% (culture MEM10) methanol, respectively, and compared with the wildtype genome (see section “Whole Genome Sequencing and Mutational Analysis”). Table 1 summarizes the mutations within each set of six isolates. Single nucleotide polymorphisms (SNP) and short deletions/insertions affected genes and intergenic regions.

**TABLE 1:** Genetic differences in isolates sampled at various time points of the adaptation of *M. extorquens* TK 0001 to high methanol concentrations.

**GO label**	**Description of the mutated gene/locus**	**Mutation location**	**Mutation type**	**Distribution of the identified adaptive mutations**			
				**5%**	**7%**	**8%**	**10%**
				**182G**	**292G**	**549G**	**855G**
TK 0001_v2_0071	Glycosyl transferase	Genic	ins -/C				1/6
TK 0001_v2_3055/TK 0001_v2_3056	Putative type I secretion system ATPase/*pufC*, photosynthetic reaction center cytochrome c subunit-related	Intergenic	snp				5/6
TK 0001_v2_3350	*metY*, *O*-acetylhomoserine sulfhydrylase	Genic	snp	•	•	•	•
TK 0001_v2_3504/TK 0001_v2_3505	*gapA*, glyceraldehyde-3-phosphate dehydrogenase/conserved protein of unknown function	Intergenic	snp		4/6	4/6	∙
TK 0001_v2_3606	*ybhL*, conserved protein of unknown function	Genic	snp				1/6
TK 0001_v2_3681	ATP-binding region, ATPase-like	Genic	snp	•	•	•	•
TK 0001_v2_3750	*rpsG*, 30S ribosomal protein S7	Genic	snp				1/6
TK 0001_v2_3750	*rpsG*, 30S ribosomal protein S7	Genic	snp				•
TK 0001_v2_3751	*rpsL*, 30S ribosomal protein S12	Genic	snp			2/6	•
TK 0001_v2_4081	*shc*, squalene-hopene cyclase	Genic	snp				1/6
TK 0001_v2_4086	*dxs*, 1-deoxyxylulose-5-phosphate synthase	Genic	del T/-	•	•	•	•
TK 0001_v2_4698	Putative CelB-like protein (fragment)	Genic	snp			5/6	•
TK 0001_v2_4727	Conserved exported protein of unknown function	Genic	snp			1/6	•
		
		**Total mutations**		**3**	**4**	**7**	**13**
		**Common mutations**		**3**	**3**	**3**	**8**

*Isolates were obtained from samples of the evolving bacterial population adapted to growth on minimal medium supplemented with 5% (MEM5), 7% (MEM7), 8% (MEM8), and 10% (MEM10) methanol. The genome of six isolates issued from each time point were sequenced and the divergences with respect to wildtype *M. extorquens* TK 0001 identified. Light orange: mutation identified at one unique time point. Medium orange: mutation identified at several time points. Dark orange: mutation found at all time points. x/6: ratio of isolates harboring the mutation. Dots indicate mutations present in all isolates at a time point. For each time point, the total number of mutations identified is indicated as well as the number of mutations common to all six isolates.*

Sequencing revealed only a small number of chromosomal mutations, which augmented over time during the adaptation. This can be attributed to the fact that each incremental adaptation to high methanol, which lasted between 200 and 300 generations, had a clonal origin, thus limiting the clonal variance of the overall adaptation. The mutations found in the intermediate strains used for subculture inoculation, i.e., G4105, G4201, and G4363, were all preserved during subsequent culturing (Table 1). Three point mutations were shared among all isolates sequenced. While the short deletion in gene *dxs* coding for 1-deoxyxylulose-5-phosphate synthase and the missense mutation in a gene coding for an ATPase like protein cannot be directly linked to methanol tolerance, the point mutation (T34M) in gene *metY* (TK 0001_3350) coding for *O*-acetyl-L-homoserine sulfhydrylase affected a locus already shown to be involved in methanol resistance. Besides the three mutations found in all isolates, additional mutations were shared among isolates obtained from subcultures grown on higher methanol. The shared mutations augmented to eight for the isolates of subculture MEM10, including missense mutations in two ribosomal genes (*rpsG* V80G, *rpsL* R94C), which might be of importance for the adaptation of these cells to the very high methanol concentration of 10%.

**FIGURE 4:**
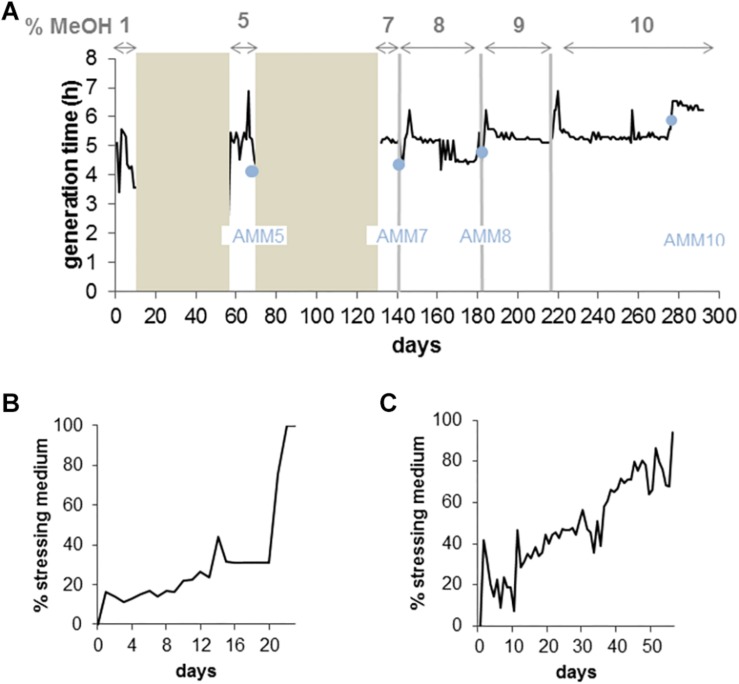
Continuous culture adaptation of *M. extorquens* AM1 bacteria to increasing methanol concentrations in a GM3 device. **(A)** Growth rate recorded for 290 days of a bacterial population cultivated on augmenting methanol concentrations. Bronze areas correspond to periods of cultivation under medium-swap regime. Black lines show the average daily generation times during cultivation periods under turbidostat regime. The methanol concentration of the culture medium applied for each evolution step (delimited by vertical gray bars) is indicated on top of the graph. Blue dots indicate time points of sampling and isolation of adaptation intermediate strains for physiology and genomic analysis. **(B)** Evolutionary kinetics of adaptive growth on minimal medium supplemented with 5% methanol of *M. extorquens* AM1 wildtype bacteria cultivated under medium-swap regime. A generation time of 8 h was set by the volume of the medium pulses injected at regular time intervals. The daily ratio of stressing medium pulses is plotted as a function of time. **(C)** Evolutionary kinetics of adaptive growth on minimal medium supplemented with 7% methanol of the 5% methanol-adapted AM1 cell population cultivated under medium-swap regime. A generation time of 7 h 30 was set. The daily ratio of stressing medium pulses is plotted as a function of time.

In a parallel experiment, we evolved the *M. extorquens* reference strain AM1 to growth on 10% methanol in the GM3 automaton. Culture regimes, isolate selection and sequencing were performed as for the TK 0001 strain. The AM1 cells, however, were evolved to high methanol resistance from a single inoculate of the wildtype strain. Figure 4 shows the evolutionary path of the AM1 cell population to growth on 10% methanol. Like the TK 0001 cells, the AM1 cell population was subjected to conditional medium swap and turbidostat adaptations. Six isolates were obtained for each of four resistance points (5, 7, 8, and 10% methanol) along the continuous culture experiment and their genomic DNA sequenced. The point mutations and short indels identified are listed in Table 2. The pattern of mutations shows a more even distribution compared to the pattern obtained for the TK 0001 isolates, where mutations accumulated upon adaptation. This difference can be attributed to the unique inoculation of the AM1 evolution. Five genes were found to be affected in all isolates obtained from the final 10% resistance culture, including gene *rpsL* (Y95C) coding for ribosomal protein S12. This locus was also affected in the TK 0001 cells adapted to very high methanol, albeit implicating a different residue (see above). As for the TK 0001 adaptation, the only gene mutated in all AM1-isolates sequenced was *metY* reinforcing the hypothesis that this locus is a hotspot of methanol-resistance. While a single mutation (T34M) was found in all TK 0001 methanol-resistant isolates, four different *metY* alleles were identified in the AM1-isolates, which were differentially distributed (Table 3).

**TABLE 2:** Genetic differences in isolates sampled at various time points of the adaptation of *M. extorquens* AM1 to high methanol concentrations.

**GO label**	**Description of the mutated gene/locus**	**Mutation location**	**Mutation type**	**Distribution of the identified adaptative mutations**			
				**5%**	**7%**	**8%**	**10%**
				**400G**	**666G**	**825G**	**1326G**
META1_0256	Glycosyl transferase	Genic	snp		•	•	•
META1_0742	Fragment of response regulator (N-terminal fragment)	Genic	snp	2/6			
META1_0744	Fragment of response regulator (C-terminal fragment)	Genic	snp	1/6			
META1_0767	Haloacid dehalogenase-like hydrolase	Genic	ins		4/6	•	•
META1_0809	Putative glycosyl transferase	Genic	ins		1/6		
META1_0953/META1_0954	*cysE*, serine acetyltransferase/*eshA*	Intergenic	snp		1/6		
META1_0984	Conserved protein of unknown function	Genic	snp	2/6			
META1_1683/ META1_1684	Putative aminoglycoside phosphotransferase/putative *O*-acyltransferase	Intergenic	snp			1/6	
META1_1766/ META1_1767	*fae*, formaldehyde-activating enzyme/orf17	Intergenic	snp			3/6	
META1_1984	Putative catecholate siderophore receptor	Genic	snp		1/6		
META1_2147	Protein of unknown function	Genic	snp				1/6
META1_2148	*rpsL*, 30S ribosomal subunit protein S12	Genic	snp		•	3/6	•
META1_2420	*clpX*, ATP-dependent Clp protease ATP-binding subunit	Genic	snp			1/6	•
META1_2508	*metY*, *O*-acetylhomoserine sulfhydrylase	Genic	snp	•	•	•	•
META1_2931	*pufB*, light-harvesting complex 1 beta chain	Genic	snp				1/6
META1_3241	*ilvA*, threonine deaminase	Genic	snp		1/6		
META1_4035	Protein of unknown function	Genic	snp	2/6			
META1_4041/ META1_4042	*rffG*, dTDP-glucose dehydratase/*rfbC*, dTDP-4-dehydrorhamnose epimerase	Intergenic	snp				1/6
META1_4735	MFS transporter membrane protein	Genic	snp				1/6
META1_5014	*mrp*, antiporter inner membrane protein	Genic	snp	•			
META1_5090/ META1_5091	*mraW*, S-adenosyl-dependent methyltransferase/RNaseP	Intergenic	snp	2/6			
META2	*grpE*, protein GrpE, Hsp cofactor / fragment of transposase of ISMex11, IS3	Intergenic	snp		1/6		
p2META_0010	Conserved protein of unknown function	Genic	snp		1/6		
		
		**Total mutations**		**7**	**10**	**7**	**9**
		**Common mutations**		**2**	**3**	**3**	**5**

*Isolates were obtained from samples of the evolving bacterial population adapted to growth on minimal medium supplemented with 5% (AMM5), 7% (AMM7), 8% (AMM8), and 10% (AMM10) methanol. The genome of six isolates issued from each time point were sequenced and the divergences with respect to wildtype *M. extorquens* AM1 identified. Light orange: mutation identified at one unique time point. Medium orange: mutation identified at several time points. Dark orange: mutation common to all time points. x/6: ratio of isolates harboring the mutation at a time point. Dots indicate mutations present in all isolates at a time point. For each time point, the total number of mutations identified is indicated as well as the number of mutations common to all six isolates.
*

**TABLE 3:** Mutations in MetY identified in the isolates analyzed at each step of the adaptation to increasing concentrations of methanol of both *M. extorquens* strains TK 0001 and AM1.

***M. extorquens* strain**	**MetY mutation**	**Distribution of the mutations in MetY between isolates at each evolution step**			
		**5%**	**7%**	**8%**	**10%**
TK 0001	T34M	6/6	6/6	6/6	6/6
AM1	G87S	–	5/6	6/6	6/6
	G113S	2/6	–	–	–
	D373G	1/6	–	–	–
	L389F	3/6	1/6	–	–

The cells isolated at different methanol resistance levels exhibited contrasted growth phenotypes (Figure 3B). Isolates obtained at late stages of the evolution showed best growth in the presence of high methanol, as was observed for the TK 0001 adaptation.

### Membrane Composition

The biophysical properties of biological membranes are of importance for adaptive responses of bacteria to external stressors. Solvents have been found to impact the fluidity of the cell membrane by triggering changes of the composition of fatty acids (Kabelitz et al., 2003; Roy, 2009; Oh et al., 2018). A whole-cell Fatty Acid Methyl Ester (FAME) analysis was conducted for the TK 0001 wildtype strain and for the two evolved strains G4105 and G4521 (Supplementary Figure S2A) as well as for AM1 and the two evolved strains G4609 and G4706 (Supplementary Figure S2B). No significant differences were observed between the wildtype and the evolved cells, indicating the membrane composition to be stable throughout the evolution on high methanol concentrations. Effects of solvent stressors on membrane composition are complex and depend on the organism (Huffer et al., 2011).

### Enzymatic Activity Tests of Wildtype and Mutated *O*-Acetyl-L-Homoserine Sulfhydrylase (MetY)

All intermediate and final isolates obtained from the high-methanol adaptations of *M. extorquens* strains TK 0001 and AM1 carried a missense mutation in the *metY* gene (TK 0001_3350 and META1_2508, respectively) coding for *O*-acetyl-L-homoserine sulfhydrylase. This enzyme catalyzes the synthesis of homocysteine, a precursor of methionine, from *O*-acetyl-L-homoserine and hydrogen sulfide (Yamagata, 1989), but can also produce methionine directly from *O*-acetyl-L-homoserine and methanethiol (Bolten et al., 2010; Supplementary Figure S5). The *metY* gene has been found to be implicated in methanol toxicity for *Corynebacterium glutamicum* (Leßmeier and Wendisch, 2015). In *Pichia pastoris* MetY was demonstrated to be related to the production of the toxic methionine analog methoxine (*O*-methyl-L-homoserine) occurring during growth in high methanol concentration. Methoxine can replace methionine during ribosomal protein synthesis leading to the accumulation of dysfunctional peptides (Leßmeier and Wendisch, 2015; Schotte et al., 2016).

All *metY* genes sequenced from the TK 0001 isolates coded for the MetY variant T34M. By contrast, the AM1 isolates harbored four different mutations in *metY*: G113S, D373G, L389F, and G87S (Table 3). The constraint-based alignment of the protein sequence of *M. extorquens* MetY with those of homologous MetY proteins whose *O*-acetyl-L-homoserine sulfhydrylase activity has been demonstrated (MetY of *C. glutamicum*, *Wolinella succinogenes*, *Pichia pastoris*, and *Thermus thermophilus*) reveals that the residues G87, G113 and D373 are highly conserved (Supplementary Figure S3). Comparisons with MetY of *W. succinogenes* for which a crystal structure has been obtained (PDB 3RI6, Tran et al., 2011) suggest that the residues T34 and L389 are part of disordered loops implicated in the monomer-monomer interfaces of the tetrameric protein. Residue G87 most likely forms a hydrogen bond with the pyridoxal-phosphate cofactor of the enzyme and G113 is adjacent to Y112 which has been proposed to stabilize the cofactor in the active-site pocket.

**FIGURE 5:**
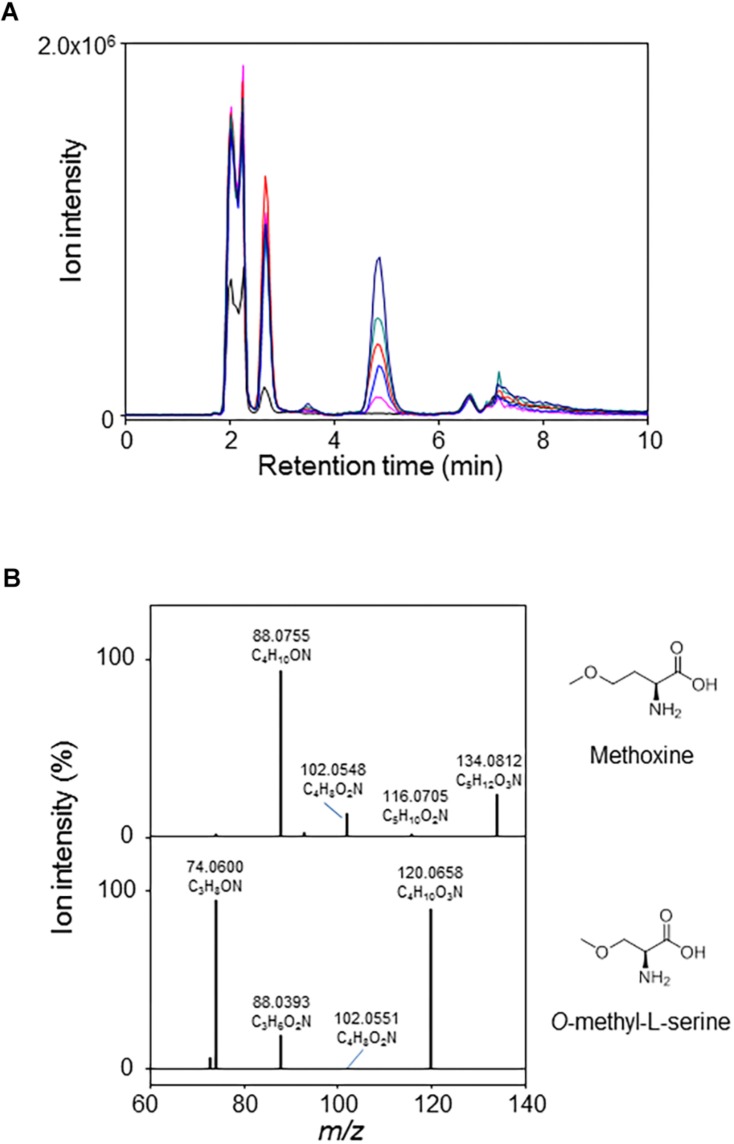
LC/MS analysis of the formation of methoxine by MetY. **(A)** Accumulation with time of methoxine in a reaction catalyzed by MetY in the presence of 3 M methanol and 5 mM *O*-acetyl-L-homoserine. Reactions were stopped and injected after 0 (black line), 5 (pink), 20 (blue), 40 (red), 60 (dark cyan), and 120 min (dark blue). Extracted Ion Chromatograms correspond to the protonated form [M+H]+ of methoxine at *m/z* 134.0812 (5 ppm accuracy). **(B)** Top: CID spectra of methoxine (at 4.8 min) in the positive mode (20% Normalized Collision Energy) from the mixture presented in panel **(A)**. Bottom: CID spectra of a 100 μM solution of *O*-methyl-L-serine in the positive mode (18% Normalized Collision Energy). *O*-methyl-L-serine is detected at m/z 120.0658 (+ 2.2 ppm off the theoretical mass).

The *metY* wildtype gene along with the five mutated alleles were cloned for recombinant protein production in *E. coli*. The kinetic parameters determined for the wildtype enzyme confirmed its activity as *O*-acetyl-L-homoserine sulfhydrylase (Table 4). No activity was found with *O*-succinyl-homoserine. Methanol could replace sulfide as nucleophilic substrate, albeit with very low affinity. We performed a LC/MS analysis of the product formed by MetY in the presence of *O*-acetyl-L-homoserine and methanol. A compound accumulating with time was detected at 4.8 min in the positive ionization mode at *m/z* 134.0812 (Figure 5A). Its mass was consistent with the monoisotopic mass of the protonated form of methoxine with an elemental composition of C_5_H_12_O_3_N^+^ (−0.1 ppm off the theoretical mass). Fragmentation spectra were recorded under collision induced dissociation (CID), and reported in Figure 5B. They displayed three main product ions: a major one at *m/z* 88.0755 relative to the loss of [CO +H_2_O], a slightly less abundant one at *m/z* 102.0548 (CH_3_OH loss) and a minor ion at *m/z* 116.0705 (H_2_O loss). In the absence of a reference compound, we compared the fragmentation spectrum of the putative methoxine with that of *O*-methyl-L-serine, a structural analog. Results showed a very close fragmentation pattern with the same losses [CO +H_2_O], [CH_3_OH], and [H_2_O] and relative intensities of the product ions (Figure 5B). Together, these results are in strong agreement with the formation of methoxine by MetY.

**TABLE 4:** Kinetic parameters of *metY*-encoded wildtype *O*-acetyl-L-homoserine sulfhydrylase of *M. extorquens* TK 0001.

**Substrate**	***k*_cat_ (s^–1^)**	***K*_M_ (mM)**	***k*_cat_/*K*_M_ (s^–1^.M^–1^)**
*O*-acetyl-L-homoserine	2.32 ± 0.12	1.30 ± 0.20	1847.51
Sodium sulfide	1.78 ± 0.12	0.80 ± 0.20	2146.81
Methanol	0.50 ± 0.02	3150.00 ± 344.0	0.158

The *k*_cat_ for methanol being only three times less than the *k*_cat_ for sulfide, at methanol concentrations of 5% (1.2 M), i.e., in the range of the *K_M_* for methanol, the enzyme is likely to produce methoxine *in vivo*, corroborating earlier findings (see above). Conversely, all five mutant MetY variants present in the adapted cells were found to be inactive. *O*-acetyl-L-homoserine did not measurably react with sulfide or methanol in the *in vitro* assays for any of the mutants. Thus by inactivating the enzyme, the mutations would also abolish the toxic side reaction with methanol *in vivo* explaining their fixation in the cultures during evolution.

### Effects of Wildtype *metY* Overexpression on Methanol Toxicity

**FIGURE 6:**
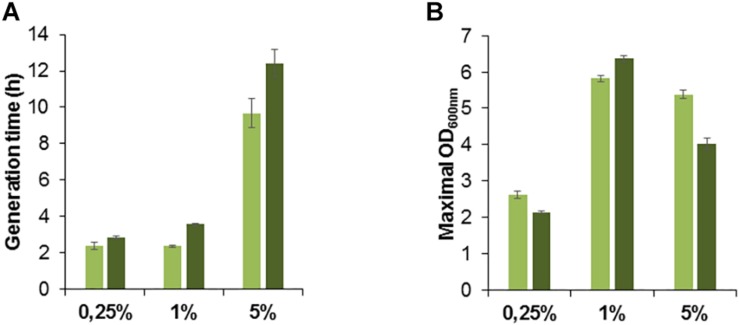
Growth of *M. extorquens* methanol adapted strain G4105 transformed with the empty vector pTE102 (light green bars) or the plasmid pTE102_*metY* (dark green bars). Generation time **(A)** and maximal OD_600__nm_ as an estimate of biomass yield **(B)** were determined after cultivation on minimal medium supplemented with 0.25, 1, or 5% methanol (v/v). Each result is the mean of triplicates; ± standard deviation is indicated.

To further assess the possible role of the *metY* locus for methanol toxicity, we inserted *metY* wildtype gene into the vector pTE102 downstream of the P*mxaF* promoter and transformed the methanol-tolerant strain G4105 with the plasmid pTE102_*metY*. We tested the resulting strain G4980 (Supplementary Table S1) for growth on different methanol concentrations. At a limiting concentration of methanol (0.25%) the G4980 cells conserved the same generation time than the cells containing an empty plasmid (Figure 6A). As expected, at higher methanol concentrations (1 and 5% (v/v)) the *metY*-expressing cells grew more slowly, with a gain in doubling time of 22% (from 9h40 to 12h25) at 5% methanol. Likewise, biomass production of G4980 cells was lowered by about 26% at this high methanol concentration as compared with the control cells (Figure 6B). These results are consistent with the implication of *O*-acetyl-L-homoserine sulfhydrylase in methanol toxicity.

### Genome-Wide mRNA Expression During Methanol Stress

We performed a transcriptome analysis to study in a systematic way the gene expression response of the *M. extorquens* TK 0001 evolved strain G4105 to exposure to high methanol and compared with the response of the ancestor wild type strain. RNA-Seq data were acquired, mapped, and normalized as described in the Section “Transcriptome Analysis.”

We determined the variations of gene expression provoked by a short-term exposure (5 min) of mid-log phase cultures grown at 1% methanol to a stressing concentration of methanol (5%) and the modulation of gene expression in cells exposed to 1 or 5% methanol on the long run (after 3 h cultivation). A threshold of log_2_(FC) greater than 2 or less than −2 was chosen as relevant expression difference for the comparisons.

The genes of *M. extorquens* TK 0001 were automatically clustered into functional categories according to the orthology framework eggNOG (Huerta-Cepas et al., 2018) operational at the MicroScope platform (Vallenet et al., 2017). Among 6160 predicted CDS in TK 0001, 3223 are associated to a functional eggNOG class (Supplementary Table S2). The remaining gene products – among them 1492 CDS listed in eggNOG class S – are without a predicted function and are not considered for the following analysis. The number of genes differentially expressed in each functional family was calculated for each pair-wise comparison.

**FIGURE 7:**
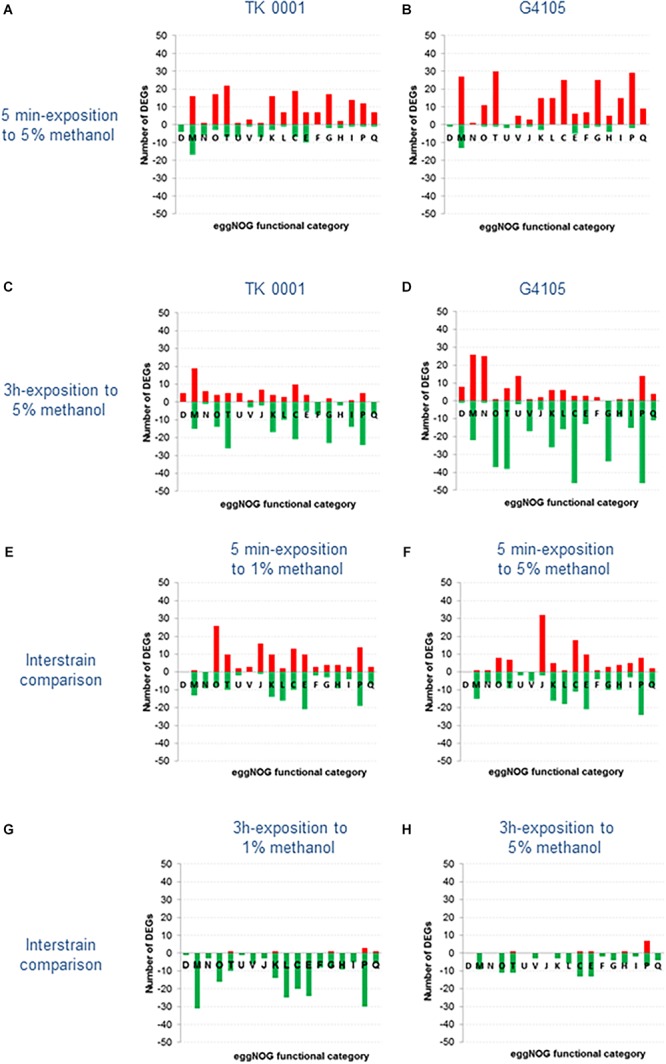
Comparative analysis of the transcriptomes of *M. extorquens* TK 0001 wildtype strain and G4105 methanol-adapted strain upon exposure to 1 and 5% methanol. Differentially expressed genes (DEGs) [log_2_(fold change) >2 or <2] were categorized based on eggNOG functional classification. Red bars represent the number of upregulated genes and green bars the number of downregulated genes in each eggNOG class. **(A)** Number of DEGs in TK 0001 cells exposed to 5% versus TK 0001 cells exposed to 1% methanol for 5 min. **(B)** Number of DEGs in G4105 cells exposed to 5% versus G4105 cells exposed to 1% methanol for 5 min. **(C)** Number of DEGs in TK 0001 cells exposed to 5% methanol for 3 h versus TK 0001 cells exposed to 5% methanol for 5 min. **(D)** Number of DEGs in G4105 cells exposed to 5% methanol for 3 h versus G4105 cells exposed to 5% methanol for 5 min. **(E)** Number of DEGs in G4105 versus TK 0001 cells, similarly exposed to 1% methanol for 5 min. **(F)** Number of DEGs in G4105 versus TK 0001 cells, similarly exposed to 5% methanol for 5 min. **(G)** Number of DEGs in G4105 versus TK 0001 cells, similarly exposed to 1% methanol for 3 h. **(H)** Number of DEGs in G4105 versus TK 0001 cells, similarly exposed to 5% methanol for 3 h.

The profiles of the pair-wise comparisons we performed are compiled in Figure 7 (for complete data set, see Supplementary Table S3). Figures 7A,B show the number of differentially expressed genes (DEGs) for each eggNOG functional category recorded for the wildtype (Figure 7A) and the adapted cells (Figure 7B) after 5 min exposure to 5% methanol. The short-term stress response resulted in similar patterns for the two strains, with 169 overexpressed genes in the wildtype and 228 in the adapted strain G4105. A higher number of overexpressed transcripts in G4105 was in particular found for categories M, T, G, and P. By contrast, the wildtype strain counted more significantly repressed genes, 80 versus 31 in G4105. The short-term high methanol exposure triggered strong overexpression (log_2_(FC) of up to 6) of a number of genes coding for certain molybdenum-binding oxidoreductases, carbohydrate mobilization enzymes, regulators of transcription and proteins maintaining DNA topology (categories F, G, K, and L). Since both strains showed comparable responses for these genes, the regulation of their expression was most likely not altered during the continuous culture adaptation process.

To study the effects of long-term exposure to high methanol on gene expression, transcriptome pair-wise analysis were conducted to compare the number of transcripts after 3 h in 5% methanol to those obtained after short term 5% methanol exposure for both TK 0001 wildtype and G4105 mutant cells (Figures 7C,D). The eggNOG functional predictions of DEGs showed that the overall expression pattern had reversed with respect to the short-term stress: functional groups dominated by either up- or downregulated genes changed the sign, with a total of 81/124 overexpressed and 191/339 repressed genes for the wildtype and the adapted strain, respectively. This was notably the case for the highly overexpressed genes mentioned above. Obviously, expression level changes induced by the 5 min methanol stress were mainly short-term responses not maintained during longer exposure to high methanol in both strains. However, some exceptions from this general observation were found for the adapted strain. Transcripts coding for the biosynthesis, assembly and functioning of flagella (category N) assuring cell motility were overexpressed in strain G4105 upon long-term high methanol exposure. Motility genes have been found to be the subject of regulation upon stress induction by a variety of chemicals including solvents (Rau et al., 2016). Likewise, genes implicated in iron homeostasis, i.e., siderophores and Fe^3+^-transporters, were strongly upregulated during prolonged growth with high methanol in the adapted strain. Since iron is a prosthetic component of proteins involved in numerous cellular processes (Andrews et al., 2003), the efficient capture of the insoluble ferric iron seems to be one of the cellular processes which played a role during directed evolution for higher methanol resistance.

Additional expression differences became apparent when the number of transcripts was directly compared between the strains. In Figure 7E are plotted the number of DEGs in each eggNOG category for strain G4105 compared to the wildtype after a 5 min growth in 1% methanol. A total of 124 genes were significantly higher, 148 were lower expressed. This result indicates that the evolved cells exhibit an altered overall physiology, which is visible also when proliferating under permissive conditions. Most affected are genes coding for heat shock proteins and chaperones (category O), among them the ATP independent small heat shock proteins (sHsp) of the *ibpA* family (Mogk et al., 2002; Haslbeck et al., 2018), the protein disaggregation chaperone ClpB (Aguado et al., 2015), and the DnaK/DnaJ proteins (Anglès et al., 2017). In addition, the ATP-dependent protease Lon which hydrolyses aberrant proteins (Bittner et al., 2016) was upregulated. The relative abundance of chaperones and proteases in the adapted cells most probably reflects the protein denaturation effect of methanol as alcoholic solvent, but might also be triggered by the accumulation of non-functional polypeptides containing methoxine instead of methionine. A large number of overexpressed genes were also found in eggNOG category J regrouping genes implicated in translation. Transcripts of ribosomal proteins constituting the 30S and 50S subunits and of chain elongation factors were upregulated. This observation might be related to a more efficient energy production from methanol in the mutant cells. A rise in the ATP pool is known to lead to an increase in ribosome numbers (Schneider et al., 2002; Pontes et al., 2016). Overexpressed genes of category C (energy production and conversion) implicated in the respiratory chain could also reflect a higher ATP production via oxidation of a surplus of NADH produced by the evolved strain.

Figure 7F shows the comparison of functional mRNA counts obtained for the adapted strain G4105 with those of the wildtype strain after growth of both strains with 5% methanol for 5 min. The total number of higher and lower expressed genes in G4105 were 106 to 171, thus comparable with the results obtained after growth with 1% methanol. However, differences between the two growth conditions were apparent, notably concerning category O (chaperones) and J (translation). Most of the chaperones and heat shock proteins were no longer overexpressed, pointing to a substantially higher expression of these genes in the wildtype strain. Obviously, the transcription increase in the wildtype cells is a response to the methanol stress, while the adapted cells produced high transcript numbers from these genes constitutively. By contrast, the number of overexpressed genes involved in translation in the adapted cells doubled to 32 upon methanol stress. The higher energy supply might indeed provoke an accelerated ribosome formation. It can be speculated that regulatory responses necessary for this short-term adaptation occurred during the strain evolution. Genes implicated in energy supply were also upregulated in strain G4105, notably subunits of the ATP synthase and of the formate dehydrogenases 1, 2, and 4 supplying the cells with reducing equivalents from methanol.

In Figures 7G,H, the comparison of the two strains is extended to the functional mRNA counts after 3 h of growth with 1% (7G) and 5% (7H) methanol. During growth at 1%, a large number of genes are higher expressed in the wildtype cells, most probably reflecting that at this methanol concentration, albeit optimal for biomass production, the wildtype cells are already challenged while the adapted G4105 cells are not. During growth at 5% for 3 h, the overall gene expression profiles converge since both strains must cope with high methanol.

### D-Lactate Production From D-Lactate Dehydrogenase Overexpressing Cells

The *M. extorquens* TK 0001 cells adapted to high methanol concentration produced more biomass from methanol than the wildtype strain when fed with 1% methanol (Figure 2B). This finding suggests that these cells, used as biotechnological production chassis, could produce chemicals from methanol with higher yield than the wildtype cells.

To verify this hypothesis, the one-step production of D-lactate from pyruvate, catalyzed by D-lactate dehydrogenase using NADH as electron donor, was tested. Pyruvate is a metabolite of the central carbon metabolism. The pool sizes of pyruvate and NADH, both ultimately produced from methanol, are susceptible to be indicators of enhanced carbon flow in the cells.

The D-lactate dehydrogenase gene *ldhA* contained in the chromosome of strain TK 0001 was amplified and cloned into plasmid pTE102 behind the promoter P*mxaF* to ensure a strong and constitutive expression. The resulting plasmid was introduced into the strains TK 0001 and G4105 to compare lactate production between wildtype and adapted cells. The baseline production of lactate was determined for the two strains harboring the empty plasmid.

**FIGURE 8:**
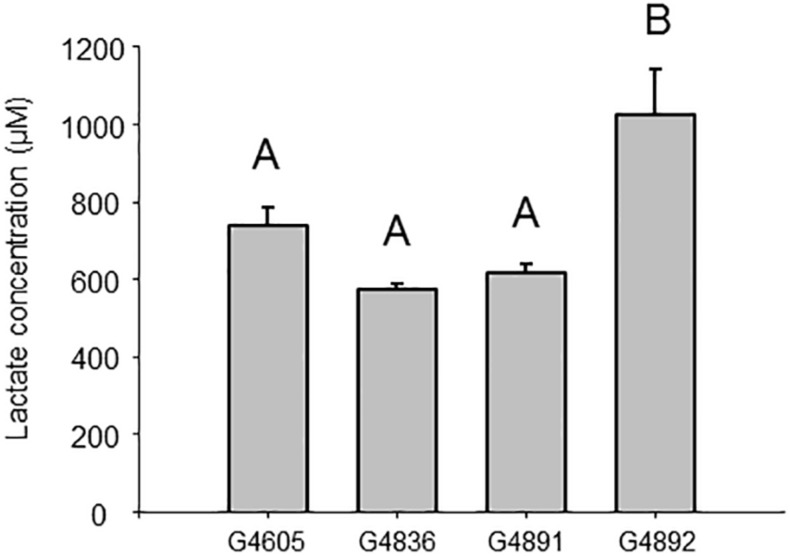
Lactate intracellular concentration (mean ± SEM) formed by G4605 (*n* = 23), G4836 (*n* = 23), G4891 (*n* = 23), and G4892 (*n* = 23). Data were normalized for 2.5 ml cell cultures at an OD_600 nm_ = 1. A non-parametric Kruskal–Wallis test followed by Dunn test was used to determine difference between groups. Bars not sharing the same letter are statistically different (*p* < 0.0083). Cellular concentrations were calculated assuming the same cell internal volume 2.310^–9^ μl for *M. extorquens* as for *E. coli* (Bazile et al., 1992).

Quantification of lactate was realized by LC-HRMS analysis, according to its EIC area in metabolomes and a calibration curve. To enable a statistical treatment of lactate production in the cells, measurements for 23 independent 2.5 ml cultures normalized at an OD_600 nm_ = 1 were conducted for each strain. Lactate amount in samples was not normally distributed (Shapiro–Wilk test). The non-parametric Kruskal–Wallis test (*p* < 0.05) allowed rejecting the null hypothesis (*p* < 0.0001). Data were then treated by the Dunn method for multiple pairwise comparisons. Results are presented in Figure 8. The intracellular amount of lactate was not different between samples from strains G4605 (TK 0001_pTE102), G4836 (G4105_pTE102), and G4891 (TK 0001_pTE102*ldhA*), but was significantly higher in sample from strain G4892 (G4105_pTE102*ldhA*) as demonstrated by statistical cross-comparison: *p* < 0.0001 between sample 4 and sample 3; *p* < 0.0001 between sample 4 and sample 2; and *p* = 0.001 between sample 4 and sample 1. The fact that the adapted cells containing plasmid pTE102*ldhA* produce more lactate from methanol than the wildtype cells harboring the same plasmid suggests an enhanced metabolic flow through the central carbon route, which is in accordance with the higher biomass yield obtained for these cells.

## Discussion

In the present study, an evolutionary approach was followed to obtain high methanol tolerant derivatives of two closely related methylotrophic strains, *M. extorquens* TK 0001 and AM1. For these strains, methanol serves as the sole carbon and energy source, but inhibits growth at concentrations above 1% (v/v). This limitation challenges the suitability of these organisms as production strains in industrial methanol fermentations.

The GM3 technology of continuous culture was used to perform the stepwise adaptation of cultures of the two strains to grow with up to 10% methanol. Medium swap culture regimes were employed to select for higher methanol tolerance. This regime enables an incremental increase of the concentration of a stressing compound in the culture. The increment depends on the enhanced growth capacities of the cells, thus maintaining a constant selection pressure. An analogous functioning is the basis of the morbidostat, a continuous culture device used to study bacterial drug resistance (Toprak et al., 2013). The medium swap regime which resembles a chemostat modified to enable the dilution of the culture by two growth media, has been found to permit lineages with different genotypes and consequently differing in fitness to prevail in the population (Marlière et al., 2011). For this reason, following a swap adaptation, the GM3 were run under turbidostat mode. During this culture phase, through selection of fastest growing cells, the populations are genetically homogenized and generally establish a stable generation time. Above 7% methanol (1.7 M), the selection of very high tolerance was performed through incrementing the methanol concentration by 1% until 10% in turbidostat. This adaptation protocol, applied to both strains, was chosen to avoid prolonged swap periods and to expose the cells to high methanol at a stable concentration.

A different inoculation strategy was pursued for the two cultures. In an attempt to accelerate the adaptation, a best growing isolate was selected at 5, 7, and 8% methanol resistance to relaunch the TK 0001 culture. By contrast, the AM1 evolution was performed from a single inoculum. However, both cultures progressed in a similar manner toward high methanol tolerance. Differences were seen for the medium swap adaptation periods (1–5%; 5–7%), which were longer for AM1, possibly reflecting the larger and more complex genome of this strain compared to TK 0001 (Belkhelfa et al., 2018).

Only three mutations were identified for the six TK 0001 isolates obtained after the 1–5% medium swap and found to be shared. No additional mutations common to all isolates analyzed were found for the adaptations to 7% in medium swap regime and the subsequent turbidostat adaptation to 8% methanol. Toward the end of the adaptation, however, the number of mutations increased cumulating to eight common mutations for the final six isolates. Higher mutation variability was found for the AM1 isolates obtained at the four resistance levels, reflecting the fact that the adaptation was performed from a single inoculum. However, the number of common mutations was limited to five for the final six isolates.

Given the strong gain in methanol resistance, the adapted isolates carried surprisingly few mutations. Possibly, the tolerance to high methanol does not require a large number of adaptive mutations, due to its relatively low potential to perturb protein structure, as compared with larger alcohols like ethanol or butanol (Perham et al., 2006). In addition, non-beneficial mutations did not accumulate due to the turbidostat selection periods where only the fittest cells stay in the culture constantly kept at exponential growth. This setup prevents mutation events to occur which are known to be specific for the stationary phase (Zambrano et al., 1993).

The *metY* gene specifying *O*-acetyl-L-homoserine sulfhydrylase carried a non-synonymous mutation in all isolates sequenced. The accumulation of loss of function mutations not affecting cell viability at this locus raises the question of the utility of this gene for the *M. extorquens* strains. As deduced from annotated genes, methionine biosynthetic routes involving trans-sulfurylation reactions are functional in both *M. extorquens* TK 0001 and AM1, what may explain the non-essential character of the MetY activity (Supplementary Figure S5). Possibly, a functional *O*-acetyl-L-homoserine sulfhydrylase widens the methionine precursor reservoir to compounds like methanethiol and dimethyldisulfide which can be used by the enzyme to directly add the terminal S-CH_3_ group to *O*-acetyl-L-homoserine to form methionine, as has been shown for MetY from *C. glutamicum* (Bolten et al., 2010). Methanol at concentrations toxic for the cells through the MetY side reaction is not likely to be found in the cell’s natural habitat, allowing the retention of the wildtype form of the enzyme.

In isolates of both evolved lineages, missense mutations were found in gene *rpsL* coding for ribosomal protein S12 (R94C in TK 0001 and Y95C in AM1). This component of the 30S subunit is known to play a crucial role in translation accuracy. A large number of *E. coli* S12 variants containing altered residues have been constructed and the decoding phenotypes tested (Agarwal et al., 2011). The mutation of residues R94 and Y95 caused a restrictive phenotype increasing the accuracy of translation. Most probably, growth in high methanol media caused increasing translational misreading leading to the selection of these mutations to lower the error rate. Solvent stressors have been described to diminish the fidelity of the ribosomal polypeptide synthesis *in vitro* (Phoenix et al., 1983). Furthermore, genes of ribosomal proteins carried mutations in *E. coli* cells adapted to high ethanol (Haft et al., 2014).

It is also noteworthy that no mutations occurred in the genes coding for proteins of formaldehyde detoxification. This is in accordance with growth phenotypes of the methanol tolerant strain G4105 on formaldehyde, which was not significantly altered with respect to the wildtype strain (Supplementary Figure S4).

Besides chromosomal mutations, evolution of microorganisms to resist solvent stressors commonly provokes alterations of gene expression suggesting adaptive mechanisms at the level of transcription (Reyes et al., 2013). The continuous culture adaptation caused broad effects on the transcription of genes involved in multiple cellular functionalities, revealed by pairwise comparisons between the wildtype and the evolved strain. Chaperones and proteases implicated in the maintenance of protein homeostasis were upregulated in the mutant, as was the case for mRNAs of ribosomal proteins. The high abundance of these genes in the adapted cells was apparent when the transcriptomes of cells grown at permissive conditions were compared, probably reflecting the better growth and higher biomass production of the mutant strain at 1% methanol. After a 5 min growth period with 5% methanol, inter-strain comparison revealed an additional increase in the number of higher expressed genes coding for ribosomal proteins in the mutant. Obviously, the adaptation to high methanol enabled the cells to form more ribosomes upon an increased supply of methanol, their sole carbon and energy source. Concomitantly, formate dehydrogenases were also overexpressed, likely to provide the energy necessary for the enhanced biomass production.

No significant overexpression [log_2_(FC) greater than 2 or less than −2] was noted for enzymes catalyzing the assimilation of methanol. It was observed that the corresponding genes, like *mxaF* coding for methanol dehydrogenase or *fae* coding for formaldehyde activating enzyme, were among the most highly expressed in both strains for all growth conditions tested, suggesting that significant upregulation of these transcripts was not feasible for the cells. However, nearly all enzymes of the central carbon route were found to be moderately overexpressed in the adapted cells [log_2_(FC) between 0.9 and 2, Supplementary Table S4]. Therefore, enhanced carbon flux and energy production through the central metabolism is a likely adaptation consequence. Evolved cells overexpressing D-lactate dehydrogenase from a plasmid grown in 1% methanol did produce more D-lactate than wildtype control cells, showing that the methanol tolerant cells can provide a genetic context for industrial production of chemicals through implemented synthetic pathways.

## Data Availability

The datasets generated for this study can be found in EBI, PRJEB27428, PRJEB29054, and PRJEB29055.

## Author Contributions

SB performed most of the experiments and wrote the manuscript. DR, KL, AP, ED, AP, and VB analyzed the data. ID, VAD, AB, LC, and EP provided the expert technical assistance. MS contributed to the experimental strategy. MB analyzed the data and wrote the manuscript. VD conceived the study and wrote the manuscript.

## Conflict of Interest Statement

The authors declare that the research was conducted in the absence of any commercial or financial relationships that could be construed as a potential conflict of interest.

## Footnotes

1 https://www.dsmz.de/services/services-microorganisms/identification-/ analysis-of-cellular-fatty-acids.html

2 http://www.polyomics.gla.ac.uk/assets/downloads/MSMetabolomicsPrepCells-Aug2013.pdf
