# Supplementary material for: Continuous Culture Adaptation of Methylobacterium extorquens AM1 and TK 0001 to Very High Methanol Concentrations
Source: Front Microbiol. 2019 Jun 20;10:1313. doi: 10.3389/fmicb.2019.01313 (PMC6595629; doi:10.3389/fmicb.2019.01313)
Supplement: Supplementary file 1 [file Image_1.pdf]

Supplementary Materials

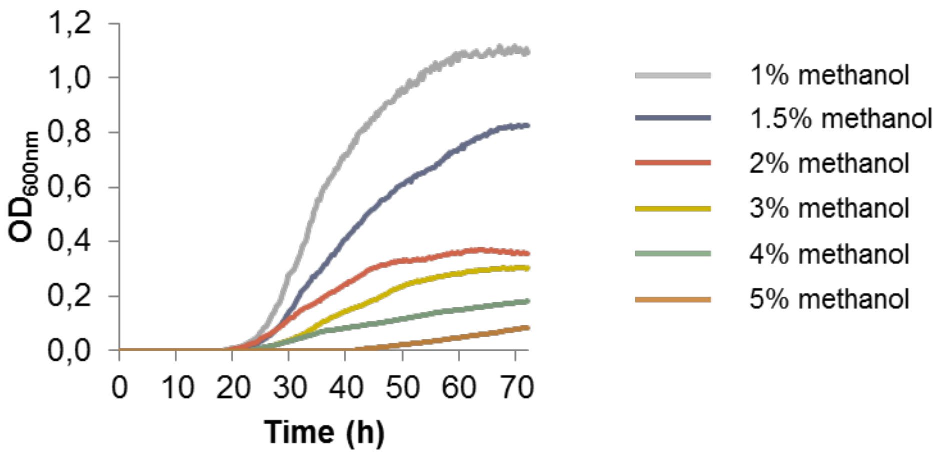

Figure S1. Growth of *M. extorquens* TK 0001 with various methanol concentrations. Growth experiments were performed on SM medium supplemented with methanol at the indicated concentrations (v/v) using a Bioscreen plate reader. Each growth curve is the mean value of 6 replicates.

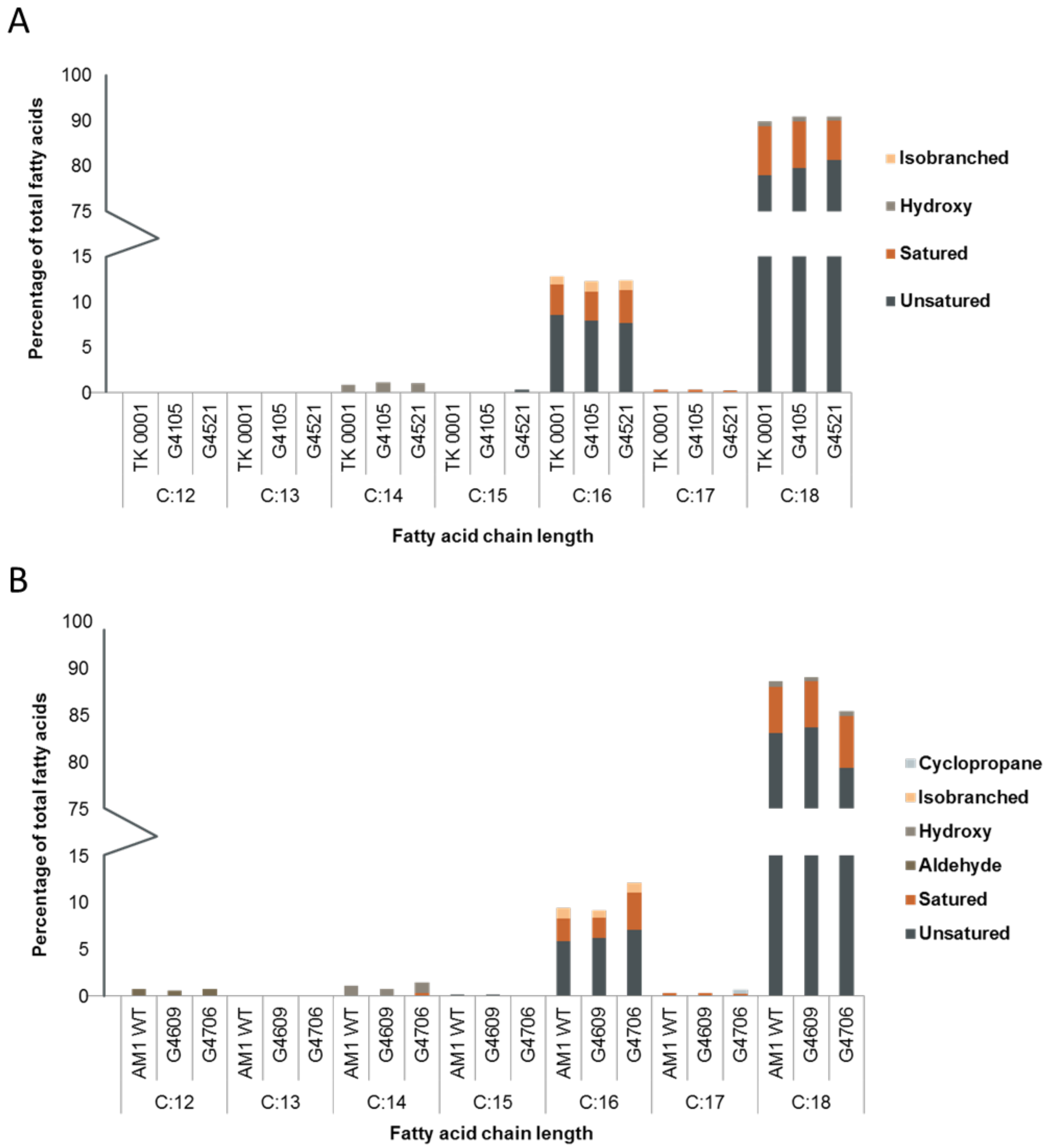

Figure S2. Whole-cell fatty acid profiles (C:12 to C:18) of wildtype and methanol-adapted *M. extorquens* strains grown on minimal medium supplemented with 1% methanol. (**A**) FAME analysis was performed on bacteria from unevolved strain *M. extorquens* TK 0001 and evolved isolates G4105 (adapted to 5 % methanol) and G4521 (adapted to 10 % methanol). (**B**) FAME analysis was performed on bacteria from unevolved strain *M. extorquens* AM1 and evolved isolates G4609 (adapted to 5 % methanol) and G4706 (adapted to 10 % methanol).

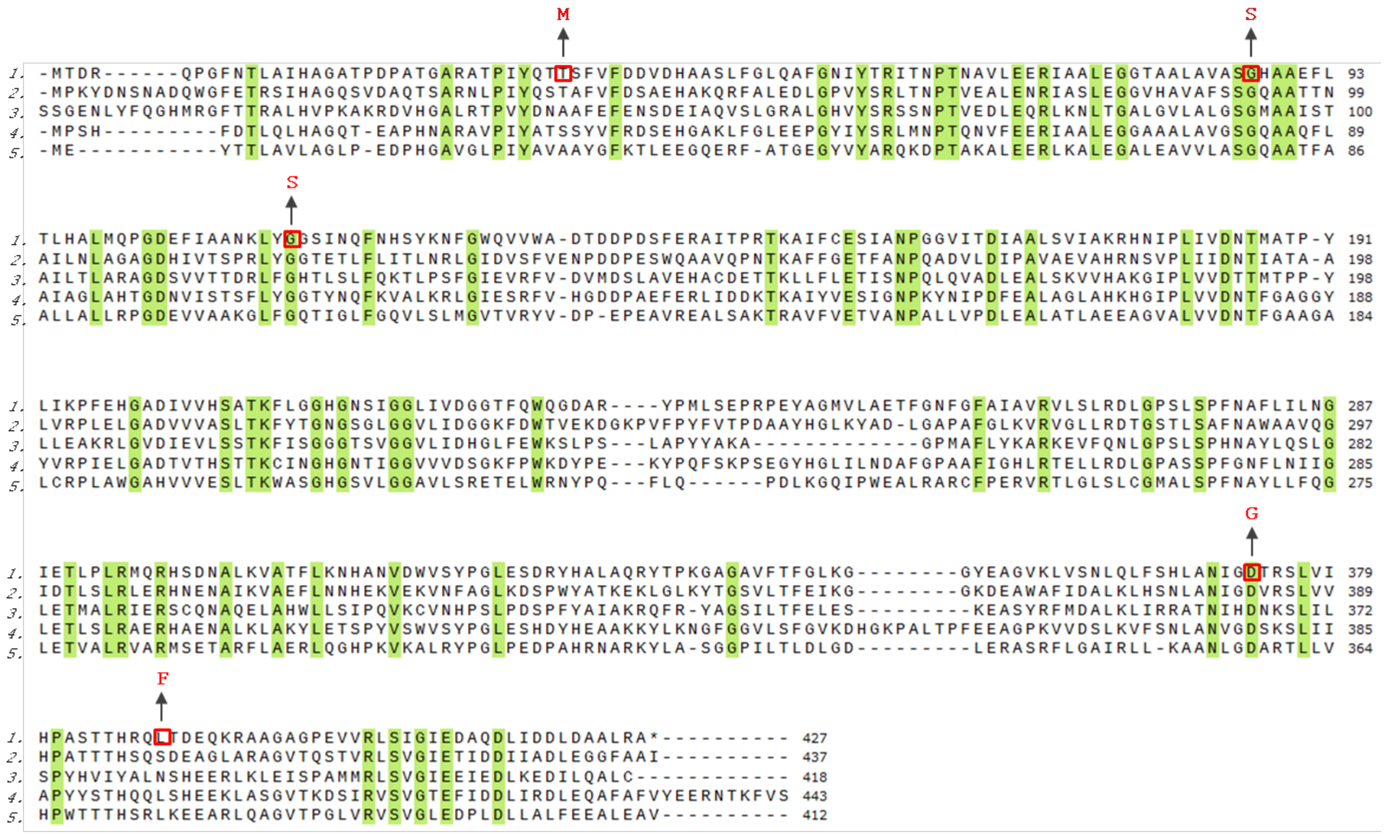

Figure S3. Sequence alignment of various MetY proteins with demonstrated *O*-acetyl-L-homoserine sulfhydrylase activity. 1. MetY *Methylobacterium extorquens* TK 0001 (TK0001_v2_3350) (this study); 2. MetY *Corynebacterium glutamicum* (CAF19359.1) (Leßmeier and Wendisch, 2015); 3. MetY *Wolinella succinogenes* (pdb 3RI6) (Tran et al, 2011); 4. MetY *Komagataella pastoris* (CAC34631.1) (Schotte et al, 2016); 5. MetY *Thermus thermophilus* (BAD02479.1) (Shimizu et al, 2001, https://doi.org/10.1016/S0167-4838(01)00245-X). The conserved residues are green highlighted. Residue changes which occurred during the TK 0001 and AM1 evolution are indicated.

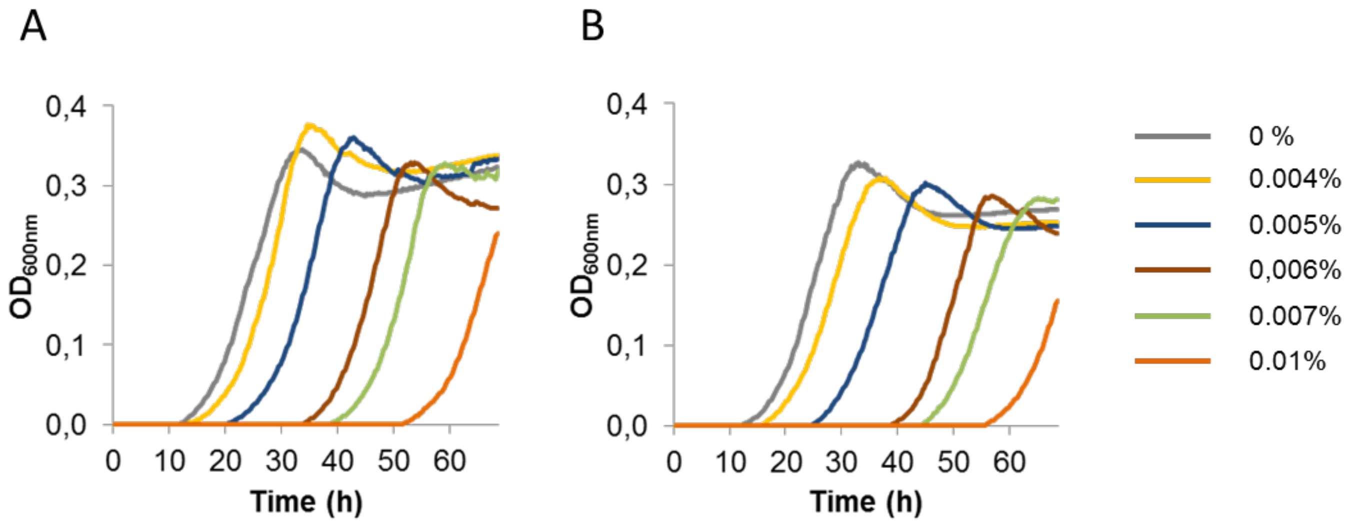

Figure S4. Growth profiles of *Methylobacterium extorquens* TK 0001 (**A**) and G4105 (**B**) on SM medium supplemented with 40 mM succinate and formaldehyde at various concentrations as indicated (v/v). Growth experiments were performed using a Bioscreen plate reader. Each result is the mean of 6 replicates.

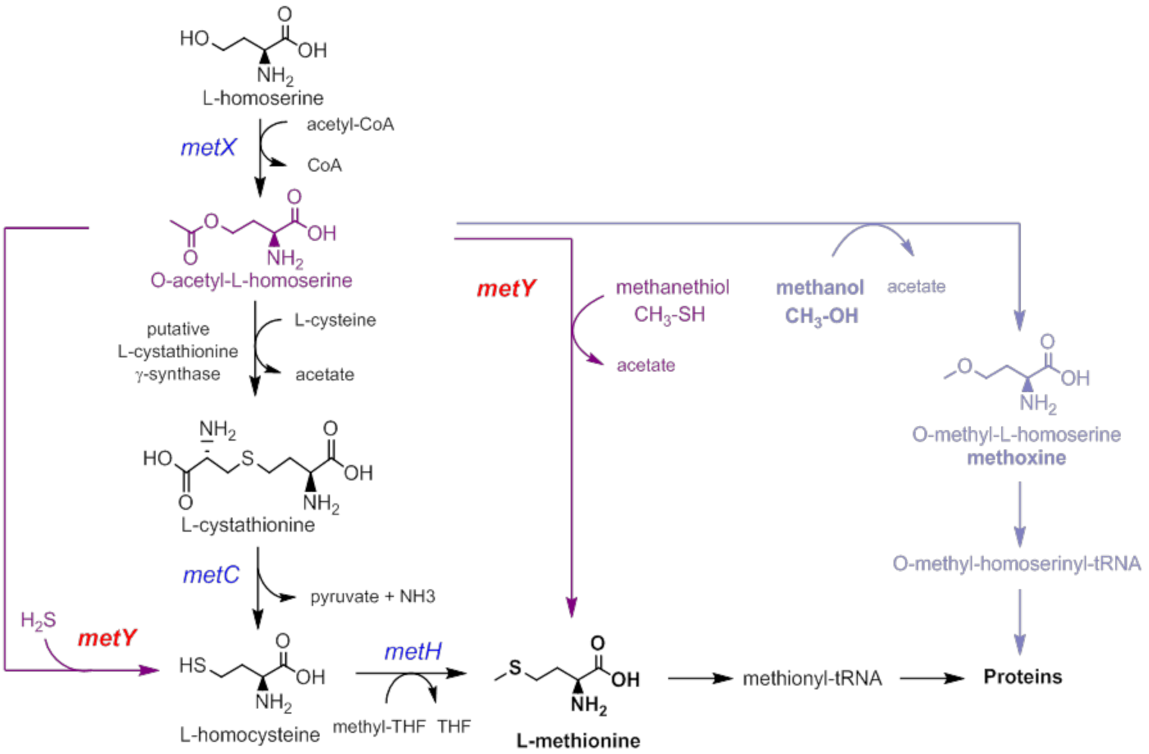

Figure S5. Methionine biosynthesis pathways of *Methylobacterium extorquens* TK 0001 and AM1 from L-homoserine as deduced from genomic annotation. *metY*-encoded *O*-acetyl-L-homoserine sulfhydrylase catalyzes the conversion of *O*-acetyl-L-homoserine into L-homocysteine using sulfide as well as the direct conversion of *O*-acetyl-L-homoserine into L-methionine using methanethiol (reactions indicated in purple). Side-activity of MetY with methanol forms methoxine (reaction indicated in mauve). *metX* codes for L-homoserine *O*-acetyltransferase, *metC* for cystathionine beta-lyase, *metH* for methionine synthase.
