# Supplementary material for: Continuous Culture Adaptation of Methylobacterium extorquens AM1 and TK 0001 to Very High Methanol Concentrations
Source: Front Microbiol. 2019 Jun 20;10:1313. doi: 10.3389/fmicb.2019.01313 (PMC6595629; doi:10.3389/fmicb.2019.01313)
Supplement: Supplementary file 2 [file Table_1.docx]

**Table S1**. Bacterial strains and plasmids

| **strain** | **description** | **EBI accession number** | **reference** |
| --- | --- | --- | --- |
| *M. extorquens* TK 0001 | wildtype |  | DSM 1337 Belkhelfa et al., 2018 |
| G4102 | derivative of wt TK 0001 adapted to growth on 5 % methanol after long-term cultivation in medium swap | ERS2573818 | this study |
| G4103 | « | ERS2573819 | « |
| G4104 | « | ERS2573820 | « |
| G4105 | « | ERS2573821 | « |
| G4106 | « | ERS2573822 | « |
| G4107 | « | ERS2573823 | « |
| G4198 | derivative of G4105 adapted to growth on 7 % methanol after long-term cultivation in medium swap | ERS2781879 | « |
| G4199 | « | ERS2781789 | « |
| G4200 | « | ERS2781778 | « |
| G4201 | « | ERS2781794 | « |
| G4202 | « | ERS2781878 | « |
| G4203 | « | ERS2781777 | « |
| G4363 | derivative of G4201 adapted to growth on 8 % methanol after long-term cultivation in turbidostat | ERS2781791 | « |
| G4364 | « | ERS2781790 | « |
| G4365 | « | ERS2781793 | « |
| G4366 | « | ERS2781792 | « |
| G4367 | « | ERS2781776 | « |
| G4368 | « | ERS2781786 | « |
| G4518 | derivative of G4363 adapted to growth on 10 % methanol after long-term cultivation in turbidostat | ERS2573824 | « |
| G4519 | « | ERS2573825 | « |
| G4520 | « | ERS2573826 | « |
| G4521 | « | ERS2573827 | « |
| G4522 | « | ERS2573828 | « |
| G4523 | « | ERS2573829 | « |
| *M. extorquens* AM1 | wildtype |  | DSM 1338 |
| G4606 | derivative of wt AM1 adapted to growth on 5 % methanol after long-term cultivation in medium swap | ERS2781831 | this study |
| G4607 | « | ERS2781830 | « |
| G4608 | « | ERS2781829 | « |
| G4609 | « | ERS2773665 | « |
| G4610 | « | ERS2781833 | « |
| G4611 | « | ERS2781832 | « |
| G4635 | derivative of wt AM1 adapted to growth on 7 % methanol after long-term cultivation in medium swap | ERS2802203 | « |
| G4636 | « | ERS2802204 | « |
| G4637 | « | ERS2802205 | « |
| G4638 | « | ERS2802206 | « |
| G4639 | « | ERS2802207 | « |
| G4640 | « | ERS2802208 | « |
| G4597 | derivative of wt AM1 adapted to growth on 8 % methanol after long-term cultivation in medium swap | ERS2802209 | « |
| G4598 | « | ERS2802210 | « |
| G4599 | « | ERS2802211 | « |
| G4600 | « | ERS2802212 | « |
| G4601 | « | ERS2802213 | « |
| G4602 | « | ERS2802214 | « |
| G4703 | derivative of wt AM1 adapted to growth on 10 % methanol after long-term cultivation in medium swap | ERS2802215 | « |
| G4704 | « | ERS2802216 | « |
| G4705 | « | ERS2802217 | « |
| G4706 | « | ERX2857349 | « |
| G4707 | « | ERS2802218 | « |
| G4708 | « | ERS2802219 | « |
| G4605 | transformation of wt TK 0001 with plasmid pTE102 |  | « |
| G4836 | transformation of G4105 with plasmid pTE102 |  | « |
| G4891 | transformation of wt TK 0001 with plasmid pTE102_*ldhA* |  | « |
| G4892 | transformation of G4105 with plasmid pTE102_*ldhA* |  | « |
| G4980 | transformation of G4105 with plasmid pTE102_*metY* |  | « |

| **plasmid** | **gene** | **reference** |
| --- | --- | --- |
| pET22(b+) | *bla* | Novagen |
| pTE102 | Tc^R^ | Addgene |
| pTE102_metY | Tc^R^ *metY M. extorquens* TK 0001 | this study |
| pTE102_ldhA | Tc^R^ *ldhA M. extorquens* TK 0001 codon-adapted for *E. coli* | « |
| pET22_metY_T34M | *bla* *metY* T34M from G4105 | « |
| pET22_metY_G87S | *bla metY* G87S from G4597 | « |
| pET22_metY_G113S | *bla metY* G113S from G4606 | « |
| pET22_metY_D373G | *bla metY* D373G from G4609 | « |
| pET22_metY_L389F | *bla metY* L389F from G4608 | « |
