# Supplementary figures and images for: Continuous Culture Adaptation of Methylobacterium extorquens AM1 and TK 0001 to Very High Methanol Concentrations
Source: Front Microbiol. 2019 Jun 20;10:1313. doi: 10.3389/fmicb.2019.01313 (PMC6595629; doi:10.3389/fmicb.2019.01313)

**Table S2.** eggNOG classification of *Methylobacterium extorquens* TK 0001 genes.


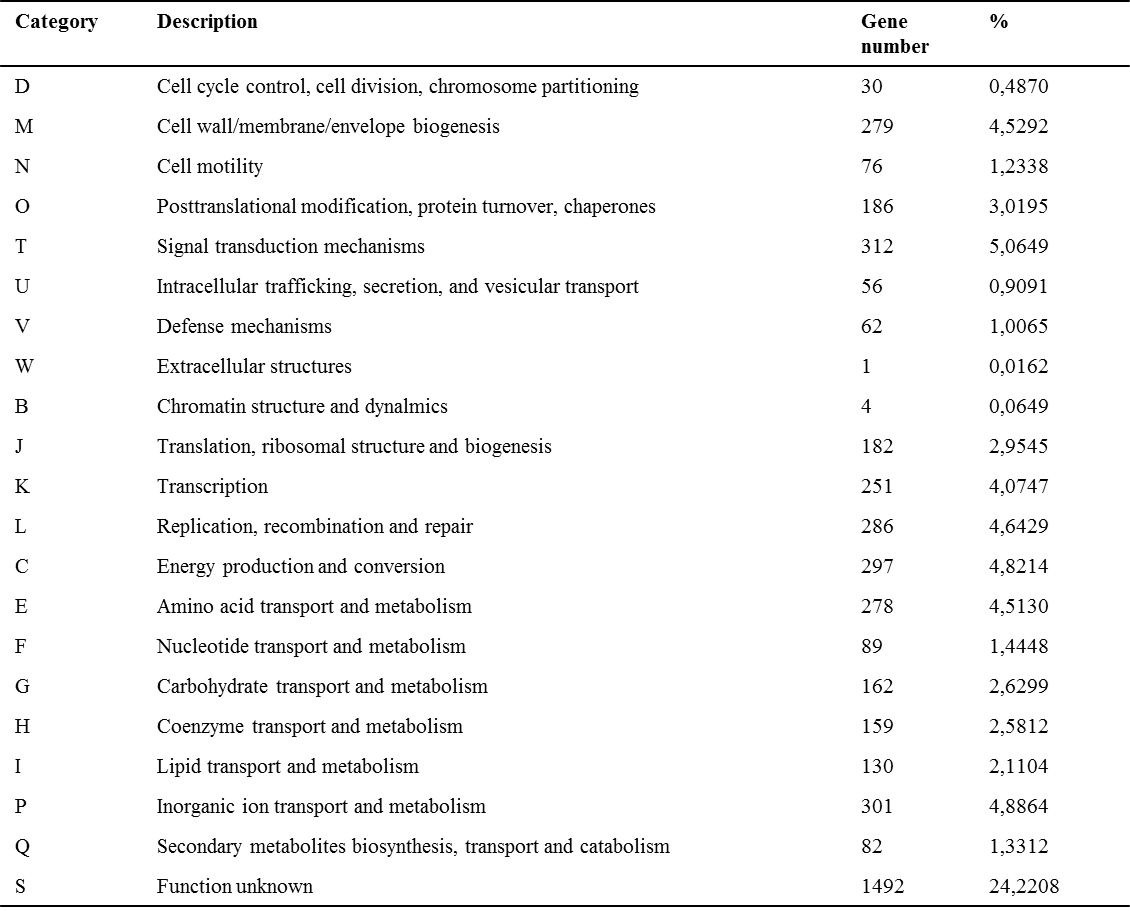

Supplement: Supplementary file 3 [file Table_2.docx]
