# Supplementary material for: Continuous Culture Adaptation of Methylobacterium extorquens AM1 and TK 0001 to Very High Methanol Concentrations
Source: Front Microbiol. 2019 Jun 20;10:1313. doi: 10.3389/fmicb.2019.01313 (PMC6595629; doi:10.3389/fmicb.2019.01313)
Supplement: Supplementary file 5 [file Table_4.docx]

**Table S4.** Differential expression of the genes implicated in methanol assimilation in the evolved strain G4105 in comparison with the ancestor strain TK 0001 upon short-term exposure to 5 % methanol.

| Gene | | GO label | Log2(FC)  (adjusted p-value < 0.05) |
| --- | --- | --- | --- |
| **Methanol oxidation** | *mxaF* | TK0001_v2_1304 | - |
|  | *mxaI* | TK0001_v2_1308 | - |
|  | *fae* | TK0001_v2_4563 | 1.12 |
|  | *mtdA* | TK0001_v2_4179 | 1.13 |
|  | *mtdB* | TK0001_v2_4145 | 1.18 |
|  | *mch* | TK0001_v2_4143 | 1.09 |
|  | *ftfL* | TK0001_v2_6037 | - |
|  | *fdh1A*  *fdh1B* | [TK0001_v2_0722](https://www.genoscope.cns.fr/agc/microscope/mage/getInfoLabel.php?id=44150720)  TK0001_v2_0723 | 1.45  0.93 |
|  | *fdh2A*  *fdh2B*  *fdh2C*  *fdh2D* | TK0001_v2_0928  TK0001_v2_0929  TK0001_v2_0930  TK0001_v2_0927 | 1.51  0.78  0.73  1.35 |
|  | *fdh3C*  *fdh3B*  *fdh3A* | TK0001_v2_6070 TK0001_v2_6071  TK0001_v2_6072 | 1.57  1.55  1.15 |
|  | *fdh4A*  *fdh4B* | TK0001_v2_3819  TK0001_v2_3820 | 1.77  1.41 |
| **Serine cycle** | *glyA* | TK0001_v2_2564 | 0.99 |
|  | *sga* | TK0001_v2_4181 | 1.18 |
|  | *hprA* | TK0001_v2_4180 | 1.04 |
|  | *gck* | TK0001_v2_3046 | 1.37 |
|  | *eno* | TK0001_v2_3004 | 1.16 |
|  | *ppc* | TK0001_v2_4175 | 1 |
|  | *mdh* | TK0001_v2_4349 | 1.02 |
|  | *mtkA* | TK0001_v2_4177 | 1.47 |
|  | *mtkB* | TK0001_v2_4176 | 1.38 |
| **Ethylmalonyl-CoA cycle** | *phaA* | TK0001_v2_2234 | - |
|  | *phaB* | TK0001_v2_2233 | - |
|  | *croR* | TK0001_v2_2260 | 1.18 |
|  | *ccr* | TK0001_v2_0049 | 2.27 |
|  | *ecm* | TK0001_v2_0045 | 1.21 |
|  | *epi* | TK0001_v2_5100 | 0.82 |
|  | *msd* | TK0001_v2_3647 | 1.47 |
|  | *meaC* | TK0001_v2_1751 | 0.91 |
|  | *mcl* | TK0001_v2_4174 | 0.88 |

-: adjusted p-value > 0.05
